# Unpacking the care‐related quality of life effect of England's publicly funded adult social care. A panel data analysis

**DOI:** 10.1002/hec.4907

**Published:** 2024-10-05

**Authors:** Andrea Salas‐Ortiz, Francesco Longo, Karl Claxton, James Lomas

**Affiliations:** ^1^ Centre for Health Economics University of York York UK; ^2^ Department of Economics and Related Studies University of York York UK

**Keywords:** adult social care, care‐related quality of life, health, instrumental variables, panel data

## Abstract

Adult Social Care (ASC) is the publicly‐funded long‐term care program in England that provides support with activities of daily living to people experiencing mental and/or physical challenges. Existing evidence suggests that ASC expenditure improves service users' care‐related quality of life (CRQoL). However, less is known about the channels through which this effect exists and the effect on outcomes other than CRQoL. We fill this gap by analyzing survey data on ASC service users who received long‐term support from 2014/15 to 2019/20 using panel data instrumental variable methods. We find that the beneficial impact of ASC expenditure on the CRQoL of both new and existing users is mostly driven by users aged 18–64 without any learning disability and users with no learning disability aged 65 or older receiving community‐based ASC. Moreover, control over daily life, occupation, and social participation are the CRQoL domains that are improved the most. We also find that ASC expenditure has a beneficial effect on several other outcomes beyond CRQoL for both new and existing users including user satisfaction and experience, the ability to carry out activities of daily living independently, whether their home is designed around needs, accessibility to local places, general health, and mental health through reduced anxiety and depression. Greater ASC expenditure, however, does not address the need for other forms of support such as unpaid informal and privately‐funded care.

## 1 INTRODUCTION

The long‐term care sector represents a substantial share of the economy. In 2017, the publicly‐funded long‐term care spending was on average 1.5% of the gross domestic product across countries in the Organization for Economic Co‐operation and Development (OECD), and about 2.2% in the United Kingdom (OECD, 2019). The publicly‐funded long‐term care program in England is called Adult Social Care (ASC). It primarily aims to improve the quality of life of individuals with mental and/or physical challenges through support with activities of daily living (Department of Health, 2006). Existing evidence (e.g., Forder et al., 2014; Forder et al., 2018; Longo et al., 2021; Longo et al., 2023a) suggests that ASC services improve service users' care‐related quality of life (CRQoL) as captured by the ASC Outcomes Toolkit (ASCOT), a policy‐relevant outcome measure designed to specifically capture the effect of long‐term care services (Netten et al., 2012). However, there is limited evidence about the channels through which ASC impacts CRQoL and the effect of ASC on outcomes other than CRQoL. Understanding these effects can inform a more complete and evidence‐based assessment of value for money. This is especially important in the context of ASC where public finance challenges are particularly acute due to increasing life expectancy and lower availability of informal carers (Ferguson and Lavalette, 2013; Foster and Harker, 2023).

Building on the analysis of service users receiving long‐term support (LTS) proposed by Longo et al. (2021, 2023, 2023), this study investigates the potential channels of the effect of ASC expenditure on the CRQoL of both new and existing LTS users (henceforth, users) by assessing whether the effect varies across some user groups. Moreover, for both new and existing users, we analyze the impact of ASC expenditure on each of the eight CRQoL domains. These capture multiple aspects of the user quality of life that are expected to be impacted by ASC services such as, for example, control over daily life, social participation, and occupation.

Our analysis then moves beyond CRQoL by focusing on new and existing users' outcomes capturing aspects of user quality of life that are unlikely to be fully captured by the CRQoL domains. These include a more qualitative measure of whether the user thinks that ASC has improved their quality of life, as well as a measure of user satisfaction and experience. As the goal for some of the ASC services that users may receive is to promote independence, we also examine the impact on the ability to carry out activities of daily living (e.g., feeding, dressing) independently. We explore as well whether ASC expenditure impacts whether surroundings help users to better cope with their needs through an improved home design or accessibility to local places. Moreover, we explore the impact of ASC expenditure on the use of unpaid informal care and privately‐funded care as improving CRQoL and independence may imply a reduced need for other forms of support. Furthermore, we test the impact of ASC expenditure on health as captured by a general health measure, as well as the pain and discomfort, and the anxiety and depression domain of the EQ‐5D questionnaire (Rabin and Charro, 2001). Finally, to analyze all these variables, we extend the cross‐sectional instrumental variable (IV) methods used by Longo et al. (2021, 2023, 2023) to panel data IV methods. These allow us to test the robustness of our results by controlling for time‐invariant unobserved heterogeneity across regions or local authorities (LAs).

The remainder of the paper is as follows. Section 1.1 provides a brief overview of the institutional background, while Section 1.2 highlights the contributions of this study in comparison with other related studies in the literature. Section 2 describes the data, and Section 3 discusses the methods. Section 4 presents the results, then discussed in Section 5. Section 6 concludes.

### 1.1 Institutional background

In England, 152 upper‐tier LAs are responsible for publicly‐funded ASC services. Access to most of these services is restricted to individuals aged 18 or older with sufficiently high needs and low finances. Eligibility criteria may vary across LAs but all LAs must guarantee a minimum level of ASC protection according to the 2014 Care Act.

ASC primarily aims to improve or maintain the quality of life of users and their (unpaid and) informal carers through a wide range of services including long‐term and short‐term support, provision of home adaptations (e.g., shower seats for disabled people), equipment (e.g., alarms for deaf people) and technology (e.g., telecare), as well as information and advice. LTS is at the core of ASC services, provided over an undefined period of time. Short‐term support is time‐limited with the aim of improving people independence and reducing their reliance on LTS. The other services tend to be provided on a one‐off basis. ASC services are delivered in the community or through institutions. Community‐based services allow people to keep living in their own homes, for example, through support from professional carers at home or befriending activities in day centres. Instead, institutional care provides users with accommodation and continuous support from professional carers in residential homes, whereas qualified nurses are also employed in nursing homes.

LAs fund ASC services using revenues from local taxes, grants from the central government and, to a lesser extent, user contributions (Amin‐Smith et al., 2018). Local taxes include council tax on the occupation of houses, and business rates tax on the occupation of businesses. Grants from the central government that are ring‐fenced for ASC are distributed across LAs according to a relative need formula, while user contributions can only be used to fund the services for which they are paid.

### 1.2 Related studies

The literature on the effects of long‐term care on service users' outcomes is growing. Some studies investigate the effect of ASC expenditure on CRQoL. One of the first studies on this topic, by Forder et al. (2014), investigates the impact of English publicly‐funded home care services for users aged 65 or older on CRQoL and finds a beneficial effect. The authors use a survey sample of 301 users and employ an IV approach using LA type dummies as instruments. There are four LA types including county, metropolitan and unitary authorities, as well as London boroughs. LAs within each type tend to share exogenous factors, such as innate culture and market factors, that are argued to determine the ASC eligibility policy and, in turn, ASC expenditure across LAs. The follow‐up study by Forder et al. (2018) explores the impact of publicly‐ and privately‐funded community‐based ASC services (e.g., home care, daycare) on CRQoL. This study also finds a beneficial effect after extending the previous analysis to all community‐based ASC services using a larger survey sample of 622 users. Unlike the previous analysis, this study employs the average level of resource use within each LA as an instrument.

The first study to investigate the effects of expenditure on all ASC services is by Longo et al. (2021) which finds a relatively small beneficial effect compared to the average CRQoL. This study uses a cross‐sectional sample of 52,602 LTS users in 2017/18 and variability in the council tax base to instrument ASC expenditure. This study also explores how the CRQoL effect varies across three groups of users: users with a learning disability,^1^ users aged between 18 and 64 years with no learning disability, and users aged 65 years or older with no learning disability. The authors find that the effect does not appear to be substantially driven by any of the three user groups. In a more recent study, Longo et al. (2023a) estimate the same effect of ASC expenditure on CRQoL but distinguish between new and existing users. The authors find that ASC improves the CRQoL of both these users, although investments in new users are more cost‐effective. They estimate a cross‐sectional model for each year from 2015/16 to 2019/20 that includes ASC expenditure and its square to allow for diminishing marginal returns. Moreover, they use cross‐sectional variation in both council tax base and LA type to address endogeneity.

The present study contributes to this literature in four ways. First, building on the analysis by Longo et al. (2023a), it pools the cross‐sectional data from 2014/15 to 2019/20 to estimate the effect of ASC expenditure on the CRQoL of new and existing users while controlling for time‐invariant unobserved heterogeneity across regions or LAs. These new specifications allow us to test the robustness of the results to various methods (cross‐sectional vs. panel data methods) and potentially consolidate the findings in the literature. Moreover, as variability in the council tax base and LA type is mostly time‐invariant across LAs, we estimate a panel IV model that controls for LA fixed effects using a novel time‐varying instrument. This captures the impact on local finances of historical choices on the council tax charge and the freeze grant scheme (Section A1 in the Appendix provides a full discussion about this instrument). Second, by analyzing more observations over multiple years, the present study can extend the analysis across user groups from three to four user groups as compared to Longo et al. (2021). In addition, we split the group of users aged 65 years or older with no learning disability by support type, that is, community‐based care and institutional (residential and nursing) care. This allows us to further examine whether the care setting explains the potential heterogeneity in the CRQoL effect on new and existing users.

Unlike all the studies discussed above, we investigate the channels generating the CRQoL effect on new and existing users as broadly defined by the eight CRQoL domains (e.g., control over daily life, social participation, etc.). An insight into how the CRQoL effect is generated through its different channels may help policymakers and ASC providers design more effective services. Finally, the present study contributes to the literature by analyzing the effect of ASC expenditure on new and existing LTS users' outcomes beyond CRQoL such as, for example, user satisfaction and experience, ability to carry out activities of daily living independently, and health. The current literature includes multiple studies that explore the impact of ASC expenditure on other outcomes. Some studies analyze the effect on health outcomes, such as population mortality (e.g., Longo et al., 2023b; Watkins et al., 2017), or hospital outcomes, such as emergency visits (e.g., Crawford et al., 2021; Liu et al., 2020), inpatient length of stay (e.g., Walsh et al., 2020), and delayed discharges (e.g., Fernandez and Forder, 2008; Gaughan et al., 2015). By assessing the impact on these broader outcomes, however, these studies cannot identify whether outcome gains or losses are experienced by service users or other individuals (e.g., healthcare patients). Therefore, by focusing on LTS users' outcomes this study aims to complement and facilitate the interpretation of the results from the existing literature.

## 2 DATA

### 2.1 Data sources

Our data come from various sources in the public domain (Table A1 of the Appendix provides all details and links). Data on users aged 18 or older receiving LTS that is funded or managed by the LA of residence are from the ASC Survey (ASCS). The ASCS is an annual survey administered by all LAs generally through the post. It collects information on users' characteristics, primary support reason, activities of daily living, generic quality of life and CRQoL, use of informal and privately‐funded care, health, and user satisfaction and experience. The ASCS uses a stratified random sample to make inferences about the survey population and four sub‐groups of users: (a) users with a learning disability, (b) users with no learning disability aged between 16 and 64, (c) users with no learning disability aged 65 or older in residential or nursing care, and (d) users with no learning disability aged 65 or older using community‐based care. The survey response rate has always been around 30% which is argued to be adequate for postal surveys (Malley and Fernandez, 2012; van Leeuwen et al., 2014). The ASCS is the only data source available for users and is commonly used for policy and research purposes (King and Wittenberg, 2015; Rand and Malley, 2017).

Data on publicly‐funded ASC expenditure and the number of users are only available at the LA level from the ASC‐Finance Return and Short and LTS database, respectively. We also collect individual data on carer characteristics from the Survey of Adult Carers in England and aggregate them at the LA‐level. LA‐level data on population characteristics are from the Census database, while data on social benefits, deprivation and local taxes come from government websites.

### 2.2 Data sample and structure

Our analysis sample includes (LTS) users within LAs from 2014/15 to 2019/20 (six financial years). These data have a multilevel structure because they include observations on users within LAs. Unlike LAs, however, the users in our sample change every year because the ASCS requests each LA to draw a different random sample every year. Therefore, in each financial year, the analysis sample is obtained after removing users who did not respond to the survey or who did so but did not respond to a question used to construct a variable in our analysis. In addition, we removed users for whom information on survey stratum, primary support reason and demographics was suppressed due to privacy reasons. On this basis, the number of users in the final sample changes depending on the variables included in the analysis.

### 2.3 User‐level variables

For each user in the final sample, we calculate the CRQoL score as measured by ASCOT. The ASCOT is a validated questionnaire (Malley et al., 2012; Rand et al., 2017) recommended for the evaluation of social care interventions in the UK (National Institute for Health Care Excellence, 2022). There are preference‐weights for ASCOT in several countries, including the UK, to weigh its eight domains: (1) control over daily life, (2) personal cleanliness and comfort, (3) food and drink, (4) personal safety, (5) social participation and involvement, (6) occupation (i.e., time spent in enjoyable activities), (7) accommodation cleanliness and comfort, and (8) dignity. Each domain has four possible responses that reflect the level of need ranging from no need to high need. The CRQoL score from ASCOT ranges between 1, the ideal state, and −0.171, with ’0′ indicating dead and negative values indicating states worse than dead. In 2019/20, for example, the CRQoL score is on average 0.818 as reported in Table 1.

**TABLE 1:** Descriptive statistics of user‐level variables.

			2014/15			2019/20		
Variable			Obs	Mean	SD	Obs	Mean	SD
CRQoL and its domains	CRQoL score		60,072	0.814	0.189	52,630	0.818	0.196
	CRQoL score for (a) users with a learning disability		13,032	0.910	0.114	14,409	0.910	0.117
	CRQoL score for (b) users with no learning disability aged between 18 and 64		12,323	0.755	0.224	9931	0.763	0.232
	CRQoL score for (c) users with no learning disability aged 65+ in residential or nursing care		9915	0.855	0.170	8306	0.850	0.177
	CRQoL score for (d) users with no learning disability aged 65+ using community‐based care		24,802	0.777	0.189	19,984	0.768	0.204
	Control over daily life score		65,986	0.110	0.051	58,551	0.111	0.051
	Personal cleanliness and comfort score		65,857	0.111	0.029	58,879	0.110	0.032
	Food and drink score		65,506	0.107	0.028	58,308	0.107	0.029
	Accommodation cleanliness and comfort score		65,534	0.108	0.020	58,750	0.108	0.022
	Safety score		65,684	0.091	0.045	58,584	0.092	0.044
	Social participation and involvement score		65,419	0.091	0.035	58,516	0.091	0.037
	Occupation (time spent in enjoyable activities) score		65,216	0.103	0.049	57,930	0.104	0.048
	Dignity score		64,413	0.091	0.034	57,178	0.091	0.035
ASC performance	Improved quality of life (by support)	ASC services helped	64,816	8%	27%	57,522	8%	27%
		ASC services did not help	64,816	92%	27%	57,522	92%	27%
	User satisfaction with support	Very	65,520	64%	48%	59,010	64%	48%
		Quite	65,520	27%	44%	59,010	25%	43%
		Neither satisfied nor dissatisfied	65,520	6%	23%	59,010	6%	24%
		Quite dissatisfied	65,520	2%	14%	59,010	2%	15%
		Vey dissatisfied	65,520	1%	12%	59,010	2%	14%
	User experience with finding information and advice	Very easy	64,395	20%	40%	56,742	45%	50%
		Fairly easy	64,395	34%	47%	56,742	17%	37%
		Fairly difficult	64,395	13%	33%	56,742	21%	41%
		Very difficult	64,395	6%	24%	56,742	11%	31%
		Never tried	64,395	27%	44%	56,742	7%	25%
Activities of daily living by themselves	Getting in and out of bed	I can do it easily	65,416	52%	50%	58,371	55%	50%
		I have difficulty doing it	65,416	24%	43%	58,371	22%	41%
		I cannot do it	65,416	24%	43%	58,371	24%	43%
	Washing face and hands	I can do it easily	65,590	69%	46%	58,501	68%	47%
		I have difficulty doing it	65,590	16%	37%	58,501	16%	37%
		I cannot do it	65,590	15%	36%	58,501	16%	36%
	Bathing and showering	I can do it easily	65,488	27%	44%	58,405	30%	46%
		I have difficulty doing it	65,488	27%	44%	58,405	26%	44%
		I cannot do it	65,488	46%	50%	58,405	44%	50%
	Using the toilet	I can do it easily	65,352	59%	49%	58,286	58%	49%
		I have difficulty doing it	65,352	18%	39%	58,286	18%	38%
		I cannot do it	65,352	23%	42%	58,286	24%	43%
	Dressing	I can do it easily	65,421	39%	49%	58,357	40%	49%
		I have difficulty doing it	65,421	27%	44%	58,357	26%	44%
		I cannot do it	65,421	34%	47%	58,357	34%	47%
	Feeding	I can do it easily	65,480	77%	42%	58,355	76%	43%
		I have difficulty doing it	65,480	16%	37%	58,355	16%	37%
		I cannot do it	65,480	8%	26%	58,355	8%	27%
	Getting around indoors	I can do it easily	65,334	50%	50%	58,190	52%	50%
		I have difficulty doing it	65,334	28%	45%	58,190	26%	44%
		I cannot do it	65,334	22%	41%	58,190	22%	42%
	Doing some paperwork	I can do it easily	65,121	20%	40%	57,947	18%	38%
		I have difficulty doing it	65,121	16%	36%	57,947	15%	36%
		I cannot do it	65,121	65%	48%	57,947	67%	47%
Surroundings and needs	Home design	Meets my needs very well	65,267	55%	50%	58,201	55%	50%
		Meets most of my needs	65,267	32%	47%	58,201	31%	46%
		Meets some of my needs	65,267	10%	30%	58,201	12%	32%
		Totally inappropriate	65,267	3%	16%	58,201	3%	17%
	Getting around outdoors	I can get to all places	64,597	29%	45%	57,427	30%	46%
		At times, I find it difficult	64,597	23%	42%	57,427	23%	42%
		I am unable to get to all places	64,597	21%	41%	57,427	21%	40%
		I do not leave my home	64,597	27%	45%	57,427	27%	44%
Other care	Unpaid informal care	Receiving this form of care	64,549	19%	39%	57,334	19%	39%
	Unpaid informal care	Not receiving this form of care	64,549	81%	39%	57,334	81%	39%
	Privately‐funded care	Receiving this form of care	63,681	63%	48%	56,035	61%	49%
	Privately‐funded care	Not receiving this form of care	63,681	37%	48%	56,035	39%	49%
Health	General health	Very good	65,671	13%	34%	58,644	16%	37%
		Good	65,671	28%	45%	58,644	27%	45%
		Fair	65,671	42%	49%	58,644	39%	49%
		Bad	65,671	13%	34%	58,644	13%	34%
		Very bad	65,671	4%	20%	58,644	5%	21%
	EQ‐5D: pain and discomfort	No	65,317	34%	47%	58,254	37%	48%
		Moderate	65,317	53%	50%	58,254	50%	50%
		Extreme	65,317	13%	34%	58,254	13%	34%
	EQ‐5D: anxiety and depression	No	64,790	48%	50%	57,554	50%	50%
		Moderate	64,790	45%	50%	57,554	42%	49%
		Extreme	64,790	8%	27%	57,554	8%	28%
User characteristics	Female		186,373	62%	49%	206,541	60%	49%
	Aged 65 or older		186,490	68%	47%	206,552	66%	47%
	Non‐white ethnicity		185,681	8%	27%	205,677	10%	30%
	Questionnaire in English		67,250	100%	3%	205,773	100%	5%
	Help with the questionnaire	No help	64,069	21%	41%	56,891	20%	40%
		Read by someone else	64,069	47%	50%	56,891	47%	50%
		Translated by someone else	64,069	18%	38%	56,891	21%	41%
		Written by someone else	64,069	40%	49%	56,891	38%	49%
		Talked through with someone else	64,069	29%	45%	56,891	30%	46%
		Answered by someone else without asking	64,069	9%	29%	56,891	10%	30%
	Easy‐read version of the questionnaire		184,169	16%	37%	206,552	18%	38%
	Primary support reason	Physical support	186,490	61%	49%	206,552	59%	49%
		Sensory support	186,490	2%	12%	206,552	1%	12%
		Support with memory and cognition	186,490	8%	27%	206,552	9%	29%
		Learning disability support	186,490	16%	36%	206,552	18%	39%
		Mental health support	186,490	12%	32%	206,552	10%	30%
		Social support	186,490	2%	13%	206,552	2%	15%

*Note*: Mean and standard deviation are weighted by the survey weight.

Abbreviations: ASC, Adult Social Care.CRQoL, Care‐Related Quality of Life; Obs, observations; SD, standard deviation.

Moreover, we measure other user‐level outcomes including whether the user thinks that ASC services have improved their quality of life (henceforth, improved quality of life indicator), overall user satisfaction with the ASC services received, and user experience in terms of difficulty in finding information and advice on services and benefits. Other user‐level variables include activities of daily living that users can carry out by themselves including being able to get in/out of bed, wash face and hands, bath or shower, use the toilet, dress, feed, get around indoors, and do some paperwork. The extent to which surroundings help users to cope with their needs is measured by asking users how well their home is designed to meet their needs and to what extent they find it difficult to access local places. We also capture whether users receive informal and privately‐funded care. Health is measured using an indicator of general health, as well as two aspects of health‐related quality of life such as the pain and discomfort domain, and the anxiety and depression domain of the EQ‐5D‐3L questionnaire.

Finally, we capture several user characteristics including whether the user is female, aged 65 or older, and of non‐white ethnicity. We also observe whether the user received a questionnaire in English, whether they received any form of help to complete the questionnaire (e.g., if the questionnaire was read or translated by someone else), and whether they received an easy‐read version (e.g., because they were visually impaired). We also collect information on their condition through the type of primary support that they receive (e.g., physical support, sensory support). Table 1 provides some descriptive statistics on all user‐level variables in 2014/15 (least recent in our sample) and 2019/20 (most recent), while the remaining 3 years are included in Tables A2 and A3.

### 2.4 LA‐level variables

A key variable constructed at the LA level is ASC expenditure per user obtained by dividing total ASC expenditure by the number of users. Total ASC expenditure includes expenditure for all ASC services activities including expenditure on LTS, short‐term support, assistive equipment, home adaptations and technologies, information and early intervention services, auxiliary ASC activities (e.g., user assessments), and commissioning and delivery services (e.g., strategic business direction). Another LA‐level variable is the proportion of users over the population aged 18 or older. We also capture informal carer characteristics including gender, age, ethnicity, employment status, and health condition.

Other LA‐level variables are population socio‐economic characteristics such as disability levels, bedroom occupancy rate, household composition, occupation, house ownership status, population density, and social benefits received (e.g., disability living allowance, income support). We also capture population deprivation in multiple dimensions simultaneously and in single dimensions separately including education, income, employment, and disability. Furthermore, we obtain the council tax base per user, where the council tax base is the standardized number of domestic properties across LAs. Finally, we construct three dummies capturing the LA type including county, metropolitan and unitary authorities (with London boroughs being the reference category). Descriptive statistics for these variables are included in Table 2.

**TABLE 2:** Descriptive statistics of local authority‐level variables.

Variable		Mean	Ov SD	Betw SD	With SD	Min	Max
Endog.	ASC expenditure per LTS user (£000)	24.178	4.594	3.740	2.680	5.686	46.390
	Prop. of LTS users	2.1%	0.5%	0.5%	0.2%	1.0%	6.0%
Instruments	Council tax base per user	26	7	7	2	12	57
	County authority	18.0%	–	–	–	0	1
	Metropolitan authority	24.0%	–	–	–	0	1
	Unitary authority	36.0%	–	–	–	0	1
	London boroughs (inner and outer)	22.0%	–	–	–	0	1
	Missing council tax revenue per user	1916	1251	1052	689	0	9002
	Business rates tax base per user	2	4	4	2	0	67
	Prop. carers who are female	68.3%	3.7%	2.7%	2.6%	44.2%	79.4%
	Prop. carers aged 65 or older	43.8%	9.3%	8.5%	4.0%	18.8%	75.6%
	Prop. carers who are white	79.6%	19.6%	17.9%	8.0%	20.3%	100.0%
	Prop. carers who are retired	56.2%	9.0%	8.5%	3.1%	25.3%	77.0%
	Prop. carers who are employed full‐time	8.8%	3.0%	2.5%	1.7%	1.4%	17.7%
	Prop. carers who are employed part‐time	10.5%	2.3%	1.7%	1.6%	0.0%	19.7%
	Prop. carers who are self‐employed full‐time	1.9%	1.1%	0.8%	0.8%	0.0%	11.5%
	Prop. carers who are self‐employed part‐time	3.1%	1.5%	1.2%	0.9%	0.0%	14.1%
	Prop. carers who are not in paid work	20.6%	5.0%	4.2%	2.7%	0.0%	40.6%
	Prop. carers who are doing voluntary work	5.6%	1.8%	1.4%	1.1%	0.0%	13.7%
	Prop. carers who are doing other type of work	6.7%	3.0%	2.8%	1.3%	0.0%	20.9%
	Prop. carers with physical impairment or disability	20.0%	3.6%	2.6%	2.6%	9.2%	50.0%
	Prop. carers with sight or hearing loss	16.2%	3.4%	2.5%	2.3%	0.0%	26.5%
	Prop. carers with mental health problems	9.1%	3.0%	2.0%	2.3%	0.0%	18.9%
	Prop. carers with a learning disability	2.8%	1.5%	1.1%	1.1%	0.0%	12.7%
	Prop. carers with long‐standing illness	27.7%	4.9%	3.1%	3.8%	0.0%	45.2%
	Prop. carers with other health conditions	13.1%	3.7%	2.5%	2.8%	0.0%	50.0%
	Prop. carers with no particular health condition	40.1%	6.0%	4.2%	4.3%	0.0%	56.5%
	Prop. people with day‐to‐day activities that are limited by a lot (ref)	8.0%	2.0%	2.0%	0.0%	4.0%	14.0%
	Prop. people with day‐to‐day activities that are limited a little	9.0%	1.0%	1.0%	0.0%	6.0%	12.0%
	Prop. people with day‐to‐day activities that are not limited	82.0%	3.0%	3.0%	0.0%	74.0%	89.0%
	Prop. people living in house with up to 0.5 persons per bedroom (ref)	14.0%	3.0%	3.0%	0.0%	5.0%	23.0%
	Prop. people living in house with over 0.5 and up to 1.0 persons per bedroom	48.0%	6.0%	6.0%	0.0%	24.0%	55.0%
	Prop. people living in house with over 1.0 and up to 1.5 persons per bedroom	22.0%	2.0%	2.0%	0.0%	10.0%	27.0%
	Prop. people living in house with over 1.5 persons per bedroom	16.0%	8.0%	8.0%	0.0%	7.0%	47.0%
	Prop. households with single persons aged 0–64	18.6%	4.4%	4.6%	0.3%	11.9%	44.4%
	Prop. households with a single person aged 65 or older	12.1%	2.1%	2.1%	0.1%	6.0%	16.7%
	Prop. people who are students or in a non‐routine occupation (ref)	82.8%	4.7%	4.7%	0.1%	72.3%	94.1%
	Prop. people who are in routine occupation	11.1%	3.4%	3.4%	0.1%	2.5%	19.7%
	Prop. people who never worked and are long‐term unemployed	6.1%	2.6%	2.7%	0.1%	2.6%	14.8%
	Prop. people who are house owners	62.9%	12.2%	12.2%	0.3%	21.7%	81.0%
	Population density (per 10,0000 individuals)	0.28	0.33	0.33	0.01	0.01	1.64
	Prop. people aged 18–64 entitled to disability support	2.1%	1.3%	0.6%	1.1%	0.1%	7.2%
	Prop. people aged 65 or older entitled to disability support	1.6%	0.8%	0.8%	0.3%	0.5%	5.4%
	Prop. people aged 65 or older claiming attendance allowance	13.8%	2.2%	2.2%	0.6%	5.9%	21.2%
	Prop. people receiving some form of income support	5.0%	1.7%	1.5%	0.8%	1.3%	11.9%
	Prop. people aged 18–64 are entitled to employment allowance	5.7%	2.0%	1.9%	0.5%	1.7%	12.9%
	Prop. people aged 18 or older entitled to personal independence payment	2.4%	1.8%	0.9%	1.5%	0.1%	9.4%
	Index of deprivation	23.040	8.130	5.450	45.910	8.090	0.970
	Index of education deprivation	21.900	8.540	3.490	48.040	8.520	0.870
	Index of income deprivation	0.150	0.050	0.030	0.330	0.050	0.010
	Index of employment deprivation	0.120	0.040	0.020	0.240	0.040	0.010
	Index of health and disability deprivation	0.080	0.640	−2.620	1.670	0.660	0.090
	East Midlands region	6.0%	–	–	–	0	1
	East of England region	7.0%	–	–	–	0	1
	South regions: London, Southeast and Southwest	44.0%	–	–	–	0	1
	Northeast region	8.0%	–	–	–	0	1
	Northwest region	15.0%	–	–	–	0	1
	West Midlands region	9.0%	–	–	–	0	1
	Yorkshire and the humber region	10.0%	–	–	–	0	1
Observations		898					
Local authorities		152					
Number of years		6					

Abbreviations: ASC, Adult Social Care; Betw, between‐group; Endog., endogenous variables; LTS, long‐term support; Ov, overall; Prop., proportion; ref, reference category; SD, standard deviation; With, within‐group.

## 3 METHODS

### 3.1 The CRQoL effect of ASC expenditure and its channels

Estimating the effect of ASC expenditure on CRQoL is challenging because expenditure is likely to be endogenous. There are two potential sources of this endogeneity: unobserved confounders and reverse causality. Unless we observe all possible determinants of CRQoL that are correlated with expenditure (e.g., all aspects of social care need), the OLS estimate of the effect of ASC expenditure on CRQoL is likely to be downwardly biased because higher (unobserved) need leads to both lower CRQoL and higher expenditure (confounding). Similarly, OLS estimates are likely to be downwardly biased because lower CRQoL may lead LAs to increase their ASC expenditure (reverse causality).

Therefore, building on the cross‐sectional model proposed by Longo et al. (2021, 2023, 2023), we estimate the following panel data IV regression:

(1) $$\text{CRQo}L_{ijkt}=\alpha_{1}+\beta_{1}\text{ex}p_{jkt}+\gamma_{1}{\left(\text{ex}p_{jkt}\right)}^{2}+\delta_{1}\text{user}s_{jkt}+\theta_{1}X_{ijkt}+\phi_{1k}+\tau_{1t}+\left(\phi_{1k}\times \tau_{1t}\right)+\varepsilon_{1ijkt},$$

where CRQoL_*ijkt*_ is the CRQoL score of user *i* (=1,…,*I*) in LA *j* (=1,…,*J*) and region *k* (=1,…,*K*) at time *t* (=2014/15,…,2019/20), *α*_*1*_ is the intercept, exp_*jkt*_ is publicly‐funded ASC expenditure per user, and users_*jkt*_ is the proportion of users; *X*_*ijkt*_ is a vector of control variables including the user‐ and LA‐level characteristics discussed in Section 2.3 and 2.4; *φ*_*1k*_ captures region fixed effects, *τ*_*1t*_ captures time fixed effects, and *ε*_*1ijkt*_ is the error term.

In Equation (1), the key marginal effect of interest of ASC expenditure on CRQoL can be calculated as *β*_*1*_+2*γ*_*1*_exp_*jt*_ for multiple levels of expenditure. If *β*_*1*_+2*γ*_*1*_exp_*jt*_>0, for example, then an increase in ASC expenditure per user increases CRQoL. This specification allows us to test whether ASC expenditure exhibits diminishing marginal returns, in which case *γ*_*1*_ < 0. Moreover, it allows us to estimate the effect of non‐marginal changes in ASC expenditure which can be of policy interest. For example, the CRQoL effect of increasing expenditure from zero to the mean level can be interpreted as providing an average ASC package to a new user. Similarly, the CRQoL effect of increasing expenditure from the minimum to the mean level can be interpreted as increasing ASC expenditure for an existing user.

Equation (1) has three endogenous variables: ASC expenditure per user, its square, and the proportion of users.^2^ To address this endogeneity, we use council tax base per user,^3^ its square, and three dummies capturing the LA type (county, metropolitan, and unitary authorities, with London boroughs being the reference category) as instruments. Following Longo et al. (2021, 2023, 2023) we argue that LAs with a larger council tax base are likely to have greater financial capacity to fund ASC services (relevance condition). In addition, conditional on the socio‐economic status across LAs, the council tax base is unlikely to be related to social care outcomes (such as CRQoL) and unobserved needs (exogeneity condition). Although wealthier populations with lower social care needs tend to live in LAs with higher council tax base, once this socio‐economic status is accounted for, the council tax base reflects the historical urban development across LAs which is independent from ASC outcomes and needs. Moreover, as CRQoL is a sector‐specific measure this is unlikely to be determined by expenditure for other local non‐ASC services that are funded through council tax (see Longo et al. (2021) for a full description of the council tax base and a longer discussion of this instrument). On the other hand, Forder et al. (2014) suggest the existence of systematic differences in ASC expenditure across LA types (relevance condition). LAs within each type share exogenous characteristics, such as innate culture and market factors, that lead to a similar ASC eligibility policy (exogeneity condition). Using these instruments, we estimate Equation (1) by the 2SLS estimator, weight observations by their survey weight, and cluster standard errors within survey strata and LAs.

As a sensitivity analysis, we include LA fixed effects in Equation (1) as follows:

(2) $$\text{CRQo}L_{ijt}=\alpha_{2}+\beta_{2}\text{ex}p_{jt}+\theta_{2}Z_{ijt}+\pi_{2j}+\tau_{2t}+\varepsilon_{2ijkt},$$

where *Z*_*ijt*_ is a vector including only the subset of controls in *X*_*ijkt*_ that vary over time across LAs, and *π*_*2j*_ captures LA fixed effects (and absorb the region fixed effects). In Equation (2), *β*_*2*_ is the key coefficient of interest which, if positive, indicates that a £1000 increase in ASC expenditure per user increases user CRQoL. The estimated *β*_*2*_ from Equation (2) can be reasonably compared with the estimated *β*_*1*_+2*γ*_*1*_exp_*jt*_ when expenditure is at the mean level. We hypothesize that *β*_*2*_ > 0 but *β*_*2*_<*β*_*1*_+2*γ*_*1*_exp_*jt*_^mean^. The latter is because, while Equation (1) analyses variability (within regions but) across LAs that may reflect a long‐run equilibrium, Equation (2) is likely to estimate a short‐run effect by analyzing contemporaneous time‐variability across LAs (e.g., Houthakker, 1965; Kuh, 1959). Moreover, since the council tax base and the LA type are mostly time‐invariant, we use variability in the amount of missing council tax revenues due to historic choices on the council tax charge and freeze grant scheme to construct a time‐varying instrument. A full discussion of this analysis is included in Section A1.

We empirically test the relevance and exogeneity of the instruments in Equations (1) and (2). In regression (1), with multiple endogenous variables and heteroscedasticity, relevance cannot be tested using the standard rule of thumb of a first‐stage F‐statistic greater than ten (Andrews et al., 2019). We, therefore, implement the more suitable two‐step procedure suggested by (Andrews, 2018).^4^ The first step of this procedure estimates the distortion in the coverage probability, that is, the probability that the true parameter of the endogenous variable is included in the estimated Wald confidence interval. Put simply, the coverage distortion is estimated by comparing the Wald confidence interval on the estimated coefficient of the endogenous variables with the weak‐instrument‐robust confidence interval obtained using a linear combination test. Following Yogo and Stock (2002), an instrument is weak if the estimated coverage distortion is greater than 10%. The second step provides the estimated weak‐instrument‐robust confidence intervals that can be used in the case of a weak instrument. We use the same test to check relevance in Equation (2). Moreover, since Equation (1) is an over‐identified IV model with three endogenous variables and five instruments, we run the Hansen‐Sargan over‐identification test to check whether our instruments are likely to be exogenous. To run the same over‐identification test for Equation (2), we use the business rates tax base per user as an additional instrument since this features some time variability.

Furthermore, using Equation (1) as our main specification, we investigate the potential channels of the CRQoL effect by analyzing whether this is driven by certain user characteristics or CRQoL domains for both new and existing users. Using the ASCS stratification (discussed in Section 2.1), we explore whether the CRQoL effects vary by whether users have a learning disability, age, and care setting. The same analysis using regression (2) is reported and discussed in Section A2. We also estimate regression (1) after replacing the CRQoL score (the dependent variable) with the preference‐weighted score for each separate CRQoL domain (discussed in Section 2.3). The effect of ASC expenditure on other outcomes.

ASC services primarily aim to increase users' quality of life. To further assess the performance of ASC in achieving this goal, we investigate the impact of ASC expenditure on an improved quality of life indicator, overall user satisfaction with the ASC services received, and user experience in terms of difficulty in finding information and advice on services and benefits. Although CRQoL and these three other measures may overlap, the latter may provide additional insight into the effect of ASC expenditure. The improved quality of life indicator may provide a simpler and more qualitative assessment of the effectiveness of ASC expenditure. Instead, user satisfaction and experience may be more indicative of the quality of the interactions between users and social care staff and facilities rather than the effectiveness.

In addition to users' quality of life, ASC services may also impact other outcomes that can be of policy interest within and outside the ASC sector. For example, ASC services that support people for a short period have the goal of increasing independence by enabling people to carry out activities of daily living independently. One example of short‐term support is community‐based vision rehabilitation that trains visually impaired people for few weeks until they are able, for instance, to do some shopping and cook by themselves (Rabiee et al., 2016). LTS users might have received or still receive short‐term support, and, on this basis, ASC expenditure may have an impact on their ability to carry out certain activities of daily living by themselves. Therefore, we explore whether ASC expenditure impacts the ability of LTS users to independently get in/out of bed, wash their face and hands, bath or shower, use the toilet, dress, feed, get around indoors, and do some paperwork. We also test whether a higher ASC expenditure translates into surroundings that help users to better cope with their needs through an improved home design or accessibility to local places (e.g., through ASC transport). More ASC support and greater user independence may address the need for other forms of support and, therefore, we also examine the effect of ASC expenditure on the use of unpaid informal and privately‐funded care.

Furthermore, by improving CRQoL, ASC may impact health (e.g., receiving LTS at home may improve mental health through less social isolation). It may also impact health more indirectly if, for example, higher ASC expenditure saves NHS resources that are reinvested in more effective treatments for LTS users (e.g., through shorter waiting times). Therefore, we estimate the effect of ASC expenditure on a measure of general health, and on two aspects of health‐related quality of life as captured by the pain and discomfort, and the anxiety and depression domain of the EQ‐5D questionnaire.

To analyze these variables, we use the same covariates and instruments in Equation (1). However, as the dependent variables discussed above are either binary or categorical, we estimate logit or ordered logit regressions by maximum likelihood and employ a control function method to address endogeneity (Wooldridge, 2015). Briefly, the control function method consists of estimating OLS residuals from the regression of each endogenous variable on the instruments and controls. These estimated residuals are then added as additional controls to the main regression. We bootstrap standard errors with 1000 replications using the repeated half‐sample bootstrap technique (Saigo et al., 2001) that takes account of clustering by survey stratum and LA, as well as survey weights.

## 4 RESULTS

Table 3 shows the key results from Equation (1), while full results are included in Table A4a. We find that, at the mean level of ASC expenditure per user (£24,178), increasing ASC expenditure per user by £1000 increases CRQoL by 0.012. This marginal effect is larger at zero and minimum ASC expenditure per user (£5686) and equal to 0.031 and 0.026, respectively. This suggests that ASC expenditure per user has diminishing marginal returns as also indicated by the negative estimated coefficient on ASC expenditure per user squared (−0.0004). All these results are statistically significant at the 1% level.^5^ Table 3 also shows the key results from regression (2) which includes LA fixed effects (full results in Table A4b). They suggest that a £1000 increase in ASC expenditure per user increases, on average, CRQoL by 0.002 (statistically significant at the 5% level). As expected, this marginal estimate is still positive but lower compared to the estimates in Equation (1) as, unlike the latter, Equation (2) is likely to estimate a short‐run effect by controlling for LA fixed effects. IV tests on Equations (1) and (2) suggest that instruments are likely to be both relevant (coverage distortion equal to 5%) and exogenous (the null hypothesis of the over‐identification test is not rejected).

**TABLE 3:** Key results from regression (1) and (2).

All users					
ASC expenditure per user	Reg (1)				Reg (2)
	Marginal	Adjusted prediction	Non‐marginal		Marginal
			New user	Existing user	
At zero	0.031***	0.2879*	0.540**	0.368**	‐
At min level (£5686)	0.026***	0.4599***			‐
At mean level (£24,178)	0.012***	0.8285***			0.002**
Observations	332,859				332,281
Coverage distortion on expenditure	5%				5%
Over‐identification test (*p*‐value)	0.227				0.454

*Note*: The adjusted predictions of Care‐Related Quality of Life are obtained using the estimated coefficient from regression (1) and the mean value of all the controls. The non‐marginal estimate for a new user is calculated as the difference between the adjusted prediction at the mean expenditure level and at zero level. The non‐marginal estimate for an existing user is obtained as the difference between the adjusted prediction at the mean expenditure level and at the minimum level. Instruments in regression (1) are the council tax base per user, its square, and county, metropolitan and unitary authority dummies. Instruments in regression (2) are the amount of missing council tax revenues per user and its square. A coverage distortion greater than 10% indicates that the instruments are overall weak. The coverage distortion in regression (1) is obtained using a grid of 1685 coefficients including 21 coefficients for ASC expenditure per user, 1 coefficient for ASC expenditure per user squared, and 78 coefficients for the proportions of users. In regression (2), the coverage distortion is obtained using an interval of 1000 coefficients for ASC expenditure per client. The over‐identification test in regression (1) can be run as the specification is over‐identified. The over‐identification test in regression (2) is run using the missing council tax revenues per user and the business rates tax base per user as instruments. Observations are weighted by the survey weight and standard errors are clustered within strata and local authorities.

Abbreviations: ASC, Adult Social Care; Marginal, marginal effect; Non‐marginal, non‐marginal effect; Reg, regression.

***p‐value<0.01, **p‐value<0.05, *p‐value<0.1.

Diminishing marginal returns of ASC expenditure per user imply that a non‐marginal increase in ASC expenditure when expenditure is zero, that is, providing a care package to a new user, is likely to be more cost‐effective compared to the same non‐marginal increase when expenditure is at a certain level, that is, increasing expenditure for an existing user. To assess cost‐effectiveness, we divide the non‐marginal change in expenditure by its corresponding SC‐QALY effect, which is calculated by assuming that the estimated CRQoL effect spans 1 year. As shown in Table 3, we find that providing a new user with an average ASC care package is likely to generate 0.540 SC‐QALY while increasing expenditure for an existing user from minimum to mean level is likely to generate 0.368 SC‐QALY. Therefore, investing in a new user has a lower cost per SC‐QALYs of £44,774 (=£5686 ÷ 0.540 SC‐QALY) compared to investing in an existing user with a cost per SC‐QALY of £50,250 (=£18,492 ÷ 0.368 SC‐QALY).

To explore the channels of these CRQoL effects, we analyze how they vary by some user characteristics and each CRQoL domain. Table 4 indicates the results from Equation (1) when estimated by survey stratum. We find that the effect of ASC expenditure per user on CRQoL is statistically significant for all groups except for (c) users with no learning disability aged 65 or older receiving residential or nursing care. Moreover, the overall cost‐effectiveness of an investment in ASC appears to be mostly driven by (b) users with no learning disability aged 18–64 as well as (d) users with no learning disability aged 65 or older receiving community‐based ASC services, rather than by (a) users with a learning disability. The SC‐QALY gains for user groups (b) and (d) are indeed substantially higher compared to (a). In turn, the cost per SC‐QALY turns out to be substantially lower for both new and existing users in (b) — £29,594 and £33,319 per SC‐QALY, respectively — and (d) — £45,024 and £50,387 per SC‐QALY — compared to (a) — £123,357 and £136,978 per SC‐QALY — and (c) — which CRQoL gains are statistically insignificant.^6^

**TABLE 4:** Key results from the analysis of user groups and CRQoL domains.

User groups												
ASC expenditure per user	User group (a)			User group (b)			User group (c)			User group (d)		
	Adjusted prediction	Non‐marginal		Adjusted prediction	Non‐marginal		Adjusted prediction	Non‐marginal		Adjusted prediction	Non‐marginal	
		New user	Existing user		New user	Existing user		New user	Existing user		New user	Existing user
At zero	0.717***	0.196**	0.135**	−0.036	0.817***	0.555***	0.716***	0.139	0.095	0.251	0.537***	0.367***
At min level (£5686)	0.778***			0.225			0.760***			0.421***		
At mean level (£24,178)	0.912***			0.780***			0.855***			0.787***		
Observations	86,827			64,428			55,301			126,303		

CRQoL domains												
ASC expenditure per user	Personal cleanliness			Accommodation cleanliness			Food and drink			Safety		
	Adjusted prediction	Non‐marginal		Adjusted prediction	Non‐marginal		Adjusted prediction	Non‐marginal		Adjusted prediction	Non‐marginal	
		New user	Existing user		New user	Existing user		New user	Existing user		New user	Existing user
At zero	0.056***	0.056***	0.038***	0.082***	0.027***	0.018***	0.054***	0.054***	0.036***	0.008	0.086*	0.059*
At min level (£5686)	0.074***			0.090***			0.072***			0.035		
At mean level (£24,178)	0.112***			0.108***			0.108***			0.094***		
Observations	367,791			366,560			364,883			367,003		

ASC expenditure per user	Social participation			Occupation			Control over daily life			Dignity		
	Adjusted prediction	Non‐marginal		Adjusted prediction	Non‐marginal		Adjusted prediction	Non‐marginal		Adjusted prediction	Non‐marginal	
		New user	Existing user		New user	Existing user		New user	Existing user		New user	Existing user
At zero	0.023	0.070**	0.048**	−0.002	0.108**	0.074**	−0.003	0.116***	0.080***	0.050***	0.042***	0.029***
At min level (£5686)	0.045**			0.032			0.033*			0.063***		
At mean level (£24,178)	0.093***			0.106***			0.113***			0.092***		
Observations	365,802			362,915			367,041			358,943		

*Note*: User group (a) = users with a learning disability, (b) = users with no learning disability aged between 16 and 64, (c) = users with no learning disability aged 65 or older in residential or nursing care, (d) = users with no learning disability aged 65 or older using community‐based care. The adjusted predictions of CRQoL are obtained using the estimated coefficient from the regression and the mean value of all the controls. The non‐marginal estimate for a new user is calculated as the difference between the adjusted prediction at the mean expenditure level and at zero level. The non‐marginal estimate for an existing user is obtained as the difference between the adjusted prediction at the mean expenditure level and at the minimum level. The controls and instruments in these regressions are the same as in (1), as well as observations are weighted by the survey weight and standard errors are clustered within strata and local authorities.

Abbreviations: ASC, Adult Social Care; CRQoL‐Care‐Related Quality of Life; Non‐marginal, non‐marginal effect.

***p‐value<0.01, **p‐value<0.05, *p‐value<0.1.

Table 4 also shows the results from Equation (1) when CRQoL is replaced with the preference‐weighted score of each of the eight CRQoL domains. We find that ASC expenditure per user has a positive and statistically significant effect on all domains except the safety domain for which the estimated effect is only weakly statistically significant (at the 10% level). Moreover, most of the SC‐QALYs of the effect of ASC expenditure per user appear to be generated through the control over daily life, occupation, and social participation domains for both new and existing users.^7^

### 4.1 Results on other outcomes

Table 5 and Table 6 show the results from a logit or ordered logit version of regression (1) when used to analyze outcomes other than CRQoL. We find that investing more in a new or existing user substantially increases the probability that the user thinks ASC has improved their quality of life (improved quality of life indicator), feels quite or very satisfied with the support received (user satisfaction), finds information and advice about services and benefits fairly or very easily (user experience). We also find that greater investments in a new or existing user are likely to increase the probability that the user becomes more independent in all aspects of the activities of daily living including getting in/out of bed, washing face and hands, bathing or showering, using the toilet, dressing, feeding, getting around indoors, and doing some paperwork. Extra investment in a new or existing user also increases the probability that the home design meets most user needs or meets user needs very well, and that the user is more able to get around outdoors either independently or not. However, for both new and existing users, increasing ASC expenditure per user does not significantly impact the probability of using other forms of support such as unpaid informal and privately‐funded care. Finally, we find that increasing ASC expenditure per user increases the probability of being in fair, good, and very good general health. Importantly, we find that ASC expenditure is likely to improve health‐related quality of life, as measured by the EQ5D questionnaire, by reducing the probability that users feel anxious and depressed. However, it does not appear to have any impact on the pain and discomfort experienced by users.

**TABLE 5:** Key results from the analysis of other outcomes (part 1).

				Non‐marginal	
Variable			Obs	New user	Existing user
ASC performance	Improved quality of life indicator (by support)	ASC services helped	360,847	0.7416***	0.4923***
	User satisfaction with the support received	Very satisfied	366,406	0.6224***	0.5411***
		Quite satisfied		0.1195***	−0.0297
		Neither satisfied nor dissatisfied		−0.1146***	−0.1730***
		Quite dissatisfied		−0.1712***	−0.1428***
		Very dissatisfied		−0.4561***	−0.1957***
	User experience in finding information and advice	Very easy	357,251	0.2158***	0.1751***
		Fairly easy		0.1565***	0.1037***
		Fairly difficult		0.025	−0.0008
		Very difficult		−0.0196	−0.0267***
		Never tried		−0.3778***	−0.2513***
Activities of daily living	Getting in and out of bed	I can do it easily	366,653	0.3439***	0.2505***
		I have difficulty doing it		0.0337	0.0028
		I cannot do it		−0.3776***	−0.2533***
	Washing face and hands	I can do it easily	367,513	0.5379***	0.4030***
		I have difficulty doing it		−0.0001	−0.0517**
		I cannot do it		−0.5378***	−0.3513***
	Bathing and showering	I can do it easily	366,993	0.2570***	0.2184***
		I have difficulty doing it		0.1847***	0.1258***
		I cannot do it		−0.4417***	−0.3442***
	Using the toilet	I can do it easily	366,099	0.4363***	0.3265***
		I have difficulty doing it		0.0373	−0.0041
		I cannot do it		−0.4737***	−0.3224***
	Dressing	I can do it easily	366,581	0.3477***	0.2862***
		I have difficulty doing it		0.1498***	0.0867***
		I cannot do it		−0.4975***	−0.3728***
	Feeding	I can do it easily	366,572	0.4961***	0.3423***
		I have difficulty doing it		−0.1292***	−0.1323***
		I cannot do it		−0.3669***	−0.2100***
	Getting around indoors	I can do it easily	365,953	0.3154***	0.2308***
		I have difficulty doing it		0.0313	0.0004
		I cannot do it		−0.3466***	−0.2312***
	Doing some paperwork	I can do it easily	364,762	0.1331***	0.1050***
		I have difficulty doing it		0.0838***	0.0600***
		I cannot do it		−0.2169***	−0.1649***

*Note*: The non‐marginal estimate for a new user is calculated as the difference between the adjusted prediction at the mean expenditure level and at zero level. The non‐marginal estimate for an existing user is obtained as the difference between the adjusted prediction at the mean expenditure level and at the minimum level. The controls and instruments in these regressions are the same as in (1), as well as observations are weighted by the survey weight and standard errors are clustered within strata and local authorities.

Abbreviations: ASC, Adult Social Care; Non‐marginal, non‐marginal effect; Obs, observations.

***p‐value<0.01, **p‐value<0.05, *p‐value<0.1.

**TABLE 6:** Key results from the analysis of other outcomes (part 2).

				Non‐marginal	
Variable			Obs	New user	Existing user
Surroundings and needs	Home design	Meets needs very well	365,702	0.4503***	0.3578***
		Meets most of my needs		0.0243	−0.0544***
		Meets some of my needs		−0.2762***	−0.2049***
		Totally inappropriate		−0.1984***	−0.0985***
	Getting around outdoors	I can get to all places	361,313	0.2794***	0.2451***
		At times I find it difficult		0.1853***	0.1361***
		I am unable to get to all places		0.1262***	0.0618***
		I do not leave my home		−0.5909***	−0.4429***
Unpaid informal care		Receiving this form of care	362,645	−0.0483	−0.0294
Privately‐funded care		Receiving this form of care	355,994	−0.0564	−0.037
Health	General health	Very good	367,944	0.1408***	0.1225***
		Good		0.2250***	0.1737***
		Fair		0.1463***	0.0463
		Bad		−0.1954***	−0.1783***
		Very bad		−0.3167***	−0.1642***
	EQ‐5D: pain and discomfort	No pain/discomfort	365,260	0.0628	0.0417
		Moderate pain/discomfort		−0.0232	−0.0168
		Extreme pain/discomfort		−0.0395	−0.0249
	EQ‐5D: anxiety and depression	Not anxious/depressed	361,983	0.4205***	0.3462***
		Moderately anxious/depressed		0.0217	−0.0959**
		Extremely anxious/depressed		−0.4422***	−0.2503***

*Note*: The non‐marginal estimate for a new user is calculated as the difference between the adjusted prediction at the mean expenditure level and at zero level. The non‐marginal estimate for an existing user is obtained as the difference between the adjusted prediction at the mean expenditure level and at the minimum level. The controls and instruments in these regressions are the same as in (1), as well as observations are weighted by the survey weight and standard errors are clustered within strata and local authorities.

Abbreviations: ASC, Adult Social Care; Non‐marginal, non‐marginal effect; Obs, observations.

***p‐value<0.01, **p‐value<0.05, *p‐value<0.1.

## 5 DISCUSSION

This study investigates the effect of ASC expenditure on the CRQoL of new and existing users and the channels through which these effects are generated using panel data methods. As in previous studies providing cross‐sectional evidence (Longo et al., 2023a), we find that ASC expenditure improves the quality of life of both new and existing users and that extra investments in new users are likely to be relatively more cost‐effective in terms of CRQoL. Moreover, new users are likely to benefit more, compared to existing users, also in terms of other outcomes including user satisfaction and experience, independence, and health.

In addition, this study explores the channels generating the CRQoL effect on new and existing users by assessing how different user groups or CRQoL domains are impacted. We find that new and existing users with no learning disability aged 18–64 in any care setting and aged 65 or older receiving care in the community are likely to benefit more compared to those with a learning disability. On the other hand, new and existing users with no learning disability aged 65 or older receiving institutional care are unlikely to experience any CRQoL gain or loss. This suggests that investing extra resources in services for users other than those that are institutionalized, in retirement age, and with no learning disability is more cost‐effective in terms of CRQoL. However, ASC services for these users not experiencing CRQoL gains may still generate benefits on other fronts and possibly be good value for money from a broader perspective. The decision to provide individuals with institutional care is generally viewed as a last resort in the presence of severe care needs due to a lack of independence and availability of adequate informal care. Although this decision might give little relief to users themselves, it may have broader impacts, for example, on adult children who were unable to guarantee adequate informal care but whose mental health and finances worsened as a result of the informal care provided. The empirical literature on informal care does indeed suggest that providing informal care may have a detrimental effect on the carer's subjective well‐being (e.g., Bobinac et al., 2010; Van den Berg et al., 2014), mental health (e.g., Heger, 2017; Schmitz and Westphal, 2015), and labor market outcomes (e.g., Fischer and Müller, 2020; Schmitz and Westphal, 2017). In addition, our findings suggest that, although ASC services impact almost all CRQoL domains of both new and existing users, control over daily life, occupation, and social participation are the three domains that are impacted the most. This result may also help explain why ASC services generate greater benefits among users who are likely to have a more active life, such as those of working age and those of retirement age but still experiencing some degree of independence (such as those receiving services in the community).

Furthermore, this study examines the effect of ASC expenditure on users' outcomes other than CRQoL. This analysis suggests that because of more ASC investments, new and existing users will be more likely to feel that ASC has helped them, to feel more satisfied, and to feel that they have become well‐linked with their LA through better information and advice. This evidence corroborates the idea that ASC services are fundamental to users and, therefore, extra investments in the current system may already substantially improve both user satisfaction and experience.

Other results suggest that ASC services can help users improve their independence with basic and instrumental activities of daily living, from feeding and dressing to doing some paperwork. Our findings also suggest that ASC may achieve greater user independence by improving home design to meet user needs (e.g., through home adaptations and equipment) and accessibility to local places (e.g., through transport services). This implies that ASC services promoting independence (e.g., short‐term support, low‐intensity LTS in the community) can potentially produce substantial net savings by addressing, at least temporarily, the need for LTS or more intensive LTS. Our results, however, also show that although greater investments in ASC may improve users' quality of life and independence, they will not lead users to give up unpaid informal care and privately‐funded care. Despite the additional ASC services, users may still have several or severe unmet needs which can only be met through other forms of care. Nevertheless, with additional ASC services, a reduction in the intensity of other forms of care is still possible, although we do not have sufficient data on intensity to evidence this potential effect.

Finally, we find that more ASC services improve general health and reduce anxiety and depression, which is in line with other studies (Chan et al., 2009; Stone et al., 2010). This suggests that extra investments in ASC may improve health through both a better health‐related quality of life and, as suggested by Martin et al. (2021) and Longo et al. (2023b), a reduction in mortality due to survival gains. Therefore, LTS users represent a channel through which the healthcare sector can generate additional health because users with better health and CRQoL will demand fewer healthcare services, which allows the NHS to offer more cost‐effective services. This provides empirical support and helps the interpretation of the findings in the literature studying the effect of ASC services on hospital outcomes (e.g., Crawford et al., 2021; Fernandez and Forder, 2008; Walsh et al., 2020).

### 5.1 Limitations

This study has some limitations. First, our estimates for new users, that is, when expenditure is zero, are obtained by extrapolation as there are no data available on the CRQoL of individuals who do not receive LTS. Moreover, Aznar et al. (2021) suggest that the survey population tends to under‐represent some sub‐groups of users in the target population including users receiving memory and cognition support, nursing care, and learning disability support within residential settings. Other user groups are under‐represented due to the survey response including users whose reason for receiving support is mental health, the youngest and oldest users, and users belonging to all ethnic minority groups (Aznar et al., 2021). Moreover, the ASCS excludes individuals that receive ASC services other than LTS such as, for example, short‐term support or equipment only. While the collection of more and better data is envisaged, the ASCS provides the best source of information at the time of writing.

## 6 CONCLUSIONS

The findings from this study contribute to the growing literature on the effect of ASC expenditure and they have some important policy implications. First, our finding that ASC impacts users' CRQoL as well as health suggests that the evaluation of ASC policies and services should consider both social care and health outcomes. Although the primary goal of ASC services is to improve CRQoL, spillover effects on health appear inevitable. On this basis, our findings also corroborate the evidence that expanding the eligibility to ASC services may be more cost‐effective in terms of quality of life and health compared to investing more in existing users. Moreover, this study suggests that investments in the ASC sector may provide better value for money in terms of CRQoL if focused on users of working age or in retirement age, but independent enough to receive support in the community. Finally, our findings suggest that ASC has the potential to improve independence and, therefore, investments in services promoting this goal may also be cost‐effective through better CRQoL and savings.

## CONFLICT OF INTEREST STATEMENT

The authors declare that they have no conflict of interest.

## ETHICS STATEMENT

This does not apply.

## PATIENT CONSENT STATEMENT

This does not apply.

## PERMISSION TO REPRODUCE MATERIAL FROM OTHER SOURCES

This does not apply.

## CLINICAL TRIAL REGISTRATION

This does not apply.

## Supporting information

Supporting Information S1

## Data Availability

All our data are in the public domain.

## ENDNOTES

^1^
Public Health England defines learning disability as a significantly reduced ability to understand new or complex information, learn new skills, and cope independently before adulthood. A person with a learning disability may also have one or more learning difficulties such as dyslexia, dyspraxia, and attention deficit hyperactivity disorder (Public Health England, 2023).

^2^
Omitting the proportion of users in our model is likely to generate a downward bias in the estimate of the effect of ASC expenditure per user on CRQoL. This is because although access to publicly‐funded ASC is restricted, as explained in Section 1.1, generosity in the eligibility policy may vary across LAs. The higher the eligibility levels, the more users receive ASC and, therefore, for a given budget, the lower the ASC expenditure for each user, that is, the lower the treatment intensity for each user. In addition, higher eligibility levels reduce users' uncertainty on future social care costs and, therefore, are expected to have a positive impact on users' CRQoL. Hence, if eligibility levels are unobserved, the effect of an increase in ASC expenditure per user on CRQoL will capture the beneficial effect on CRQoL of greater intensity of care and the detrimental effect on CRQoL of lower eligibility levels generating a downward bias.

^3^
Following Longo et al. (2021), we divide the council tax base by the number of users to replicate LAs' funding approach as closely as possible. LAs in England are expected to allocate funding by prioritizing services that must be guaranteed by law. ASC is one of these services and, therefore, LAs allocate to this sector most of the council tax revenues (Department for Communities and Local Government, 2017). The exact proportion of council tax revenues that LAs allocate to ASC is likely to be determined by the expected number of users rather than people. Moreover, as expectations on users are driven by local eligibility policy and level of need observable to LAs, once these have been accounted for, as we do, the number of users can be argued to be exogenous to LAs given the legal requirements.

^4^
We implement this procedure using the Stata command *twostepweakiv* (Sun, 2018). We test a grid of 1685 coefficients including 21 coefficients for ASC expenditure per user, 1 coefficient for ASC expenditure per user squared, and 78 coefficients for the proportions of users. In regression, we test an interval of 1000 coefficients for ASC expenditure per user.

^5^
The results of regression (1) estimated by OLS are reported in Table A4a and show that, as expected, the estimated marginal effect of ASC expenditure per user is downwardly biased.

^6^
Marginal effects at mean ASC expenditure were statistically different except for (b) users with no learning disability aged between 16 and 64 and (d) those aged 65 or older using community‐based care. These results are shown in Table A5.

^7^
Table A6 includes the results from (2) suggesting that the CRQoL effect is mostly driven by the CRQoL gains for (d) users with no learning disability aged 65 or older receiving community‐based ASC services. Moreover, time spent in enjoyable activities (occupation domain) explains most of the CRQoL effect of ASC expenditure.

## References

[hec4907-bib-0001] Amin‐Smith, N. , Phillips, D. , & Simpson, P. (2018). Adult social care funding: A local or national responsibility (Vol. BN227). Institute for Fiscal Studies.

[hec4907-bib-0002] Andrews, I. (2018). Valid two‐step identification‐robust confidence sets for GMM. The Review of Economics and Statistics, 100(2), 337–348. 10.1162/rest_a_00682

[hec4907-bib-0003] Andrews, I. , Sock, J. , & Sun, L. (2019). Weak instruments in IV regression: Theory and practice. Annual Review of Economics, 11, 727–753. 10.1146/annurev-economics-080218-025643

[hec4907-bib-0004] Aznar, C. , Blake, M. , Mackie, M. , Pickering, K. & Rehsi, A. 2021. Representativeness of adult social care surveys.

[hec4907-bib-0005] Bobinac, A. , van Exel, N. J. A. , Rutten, F. F. , & Brouwer, W. B. (2010). Caring for and caring about: Disentangling the caregiver effect and the family effect. Journal of Health Economics, 29(4), 549–556. 10.1016/j.jhealeco.2010.05.003 20579755

[hec4907-bib-0006] Chan, S. W.‐C. , Chiu, H. F. , Chien, W.‐T. , Goggins, W. , Thompson, D. , & Hong, B. (2009). Predictors of change in health‐related quality of life among older people with depression: A longitudinal study. International Psychogeriatrics, 21(6), 1171–1179. 10.1017/s1041610209990950 19781111

[hec4907-bib-0007] Crawford, R. , Stoye, G. , & Zaranko, B. (2021). Long‐term care spending and hospital use among the older population in England. Journal of Health Economics, 78, 102477. 10.1016/j.jhealeco.2021.102477 34153887

[hec4907-bib-0008] Department for Communities and Local Government . (2017). Local authority revenue expenditure and financing: 2017‐18 budget.

[hec4907-bib-0009] Department of Health . (2006). Our health, our care, our say: A new direction for community services. The Stationery Office.

[hec4907-bib-0010] Ferguson, I. , & Lavalette, M. (2013). Adult social care. Policy Press.

[hec4907-bib-0011] Fernandez, J.‐L. , & Forder, J. (2008). Consequences of local variations in social care on the performance of the acute health care sector. Applied Economics, 40(12), 1503–1518. 10.1080/00036840600843939

[hec4907-bib-0012] Fischer, B. , & Müller, K.‐U. (2020). Time to care? The effects of retirement on informal care provision. Journal of Health Economics, 73, 102350. 10.1016/j.jhealeco.2020.102350 32615361

[hec4907-bib-0013] Forder, J. , Malley, J. , Towers, A. M. , & Netten, A. (2014). Using cost‐effectiveness estimates from survey data to guide commissioning: An application to home care. Health Economics, 23(8), 979–992. 10.1002/hec.2973 24038337

[hec4907-bib-0014] Forder, J. , Vadean, F. , Rand, S. , & Malley, J. (2018). The impact of long‐term care on quality of life. Health Economics, 27(3), e43–e58. 10.1002/hec.3612 29098741 PMC5901009

[hec4907-bib-0015] Foster, D. , & Harker, R. (2023). Adult social care funding (England). Commons Library Research Briefing.

[hec4907-bib-0016] Gaughan, J. , Gravelle, H. , & Siciliani, L. (2015). Testing the bed‐blocking hypothesis: Does nursing and care home supply reduce delayed hospital discharges? Health Economics, 24(S1), 32–44. 10.1002/hec.3150 25760581 PMC4406135

[hec4907-bib-0017] Heger, D. (2017). The mental health of children providing care to their elderly parent. Health Economics, 26(12), 1617–1629. 10.1002/hec.3457 27917556

[hec4907-bib-0018] Houthakker, H. S. (1965). New evidence on demand elasticities. Econometrica: Journal of the Econometric Society, 33(2), 277–288. 10.2307/1909790

[hec4907-bib-0019] King, D. & Wittenberg, R. 2015. Data on adult social care.

[hec4907-bib-0020] Kuh, E. (1959). The validity of cross‐sectionally estimated behavior equations in time series applications. Econometrica: Journal of the Econometric Society, 27(2), 197–214. 10.2307/1909442

[hec4907-bib-0021] Liu, D. , Pace, M. L. , Goddard, M. , Jacobs, R. , Wittenberg, R. , & Mason, A. (2020). Investigating the relationship between social care supply and healthcare utilization by older people in England. Health Economics, 30(1), 36–54. 10.1002/hec.4175 33098348

[hec4907-bib-0022] Longo, F. , Claxton, K. P. , Martin, S. , & Lomas, J. (2023). More long‐term care for better health care and vice versa: Investigating the mortality effects of interactions between these public sectors. Fiscal Studies.

[hec4907-bib-0023] Longo, F. , Claxton, K. , Lomas, J. , & Martin, S. (2021). Does public long‐term care expenditure improve care‐related quality of life of service users in England? Health Economics, 30(10), 2561–2581. 10.1002/hec.4396 34318556

[hec4907-bib-0024] Longo, F. , Claxton, K. , Lomas, J. , & Martin, S. (2023). Is extending eligibility for adult social care better than investing more in existing users in England? A cross‐sectional evidence for multiple financial years. BMJ Open, 13(9), e070833. 10.1136/bmjopen-2022-070833 PMC10496707 37696632

[hec4907-bib-0025] Malley, J. N. , Towers, A.‐M. , Netten, A. P. , Brazier, J. E. , Forder, J. E. , & Flynn, T. (2012). An assessment of the construct validity of the ASCOT measure of social care‐related quality of life with older people. Health and Quality of Life Outcomes, 10(1), 21. 10.1186/1477-7525-10-21 22325334 PMC3305488

[hec4907-bib-0026] Malley, J. , & Fernandez, J.‐L. (2012). Patterns and effects of nonresponse in the English adult social care survey. Personal Social Services Research Unit, London School of Economics.

[hec4907-bib-0027] Martin, S. , Longo, F. , Lomas, J. , & Claxton, K. P. (2021). The causal impact of social care, public health and healthcare expenditure on mortality in England: Cross‐sectional evidence for 2013/14. BMJ Open, 11(10), e046417. 10.1136/bmjopen-2020-046417 PMC8559090 34654700

[hec4907-bib-0028] National Institute for Health Care Excellence . (2022). NICE health technology evaluations: The manual. [Online]. Available: https://www.nice.org.uk/process/pmg36/resources/nice‐health‐technology‐evaluations‐the‐manual‐pdf‐72286779244741

[hec4907-bib-0029] Netten, A. , Burge, P. , Malley, J. , Potoglou, D. , Towers, A.‐M. , Brazier, J. , Flynn, T. , Forder, J. J. H. T. A. , & Wall, B. (2012). Outcomes of social care for adults: Developing a preference‐weighted measure. Health Technology Assessment, 16, 1–166. 10.3310/hta16160 22459668

[hec4907-bib-0030] OECD [Organisation for Economic Co‐Operation Development] . 2019. Long‐term care spending and unit costs.

[hec4907-bib-0031] Public Health England . (2023). Guidance: Learning disability ‐ applying all our health. [Online]. Available: https://www.gov.uk/government/publications/learning‐disability‐applying‐all‐our‐health/learning‐disabilities‐applying‐all‐our‐health#:~:text=a%20significantly%20reduced%20ability%20to,disability%20is%20different%20for%20everyone. Accessed 26 Jul 2024.

[hec4907-bib-0032] Rabiee, P. , Bernard, S. , Baxter, K. , & Parker, G. (2016). Community‐based vision rehabilitation provision in England. British Journal of Visual Impairment, 34(3), 248–261. 10.1177/0264619616658313

[hec4907-bib-0033] Rabin, R. , & Charro, F. D. (2001). EQ‐5D: A measure of health status from the EuroQol group. Annals of Medicine, 33(5), 337–343. 10.3109/07853890109002087 11491192

[hec4907-bib-0034] Rand, S. , & Malley, J. (2017). The factors associated with care‐related quality of life of adults with intellectual disabilities in England: Implications for policy and practice. Health and Social Care in the Community, 25(5), 1607–1619. 10.1111/hsc.12354 27109857 PMC5574010

[hec4907-bib-0035] Rand, S. , Malley, J. , Towers, A.‐M. , Netten, A. , & Forder, J. (2017). Validity and test‐retest reliability of the self‐completion adult social care outcomes toolkit (ASCOT‐SCT4) with adults with long‐term physical, sensory and mental health conditions in England. Health and Quality of Life Outcomes, 15(1), 163. 10.1186/s12955-017-0739-0 28821303 PMC5562982

[hec4907-bib-0036] Saigo, H. , Shao, J. , & Sitter, R. R. (2001). A repeated half‐sample bootstrap and balanced repeated replications for randomly imputed data. Survey Methodology, 27, 189–196.

[hec4907-bib-0037] Schmitz, H. , & Westphal, M. (2015). Short‐and medium‐term effects of informal care provision on female caregivers’ health. Journal of Health Economics, 42, 174–185. 10.1016/j.jhealeco.2015.03.002 25968112

[hec4907-bib-0038] Schmitz, H. , & Westphal, M. (2017). Informal care and long‐term labor market outcomes. Journal of Health Economics, 56, 1–18. 10.1016/j.jhealeco.2017.09.002 28946010

[hec4907-bib-0039] Stone, A. A. , Schwartz, J. E. , Broderick, J. E. , & Deaton, A. 2010. A snapshot of the age distribution of psychological well‐being in the United States. Proceedings of the National Academy of Sciences, 107, 9985–9990. 10.1073/pnas.1003744107 PMC2890490 20479218

[hec4907-bib-0040] Sun, L. (2018). Implementing valid two‐step identification‐robust confidence sets for linear instrumental‐variables models. STATA Journal, 18(4), 803–825. 10.1177/1536867x1801800404

[hec4907-bib-0041] Van den Berg, B. , Fiebig, D. G. , & Hall, J. (2014). Well‐being losses due to care‐giving. Journal of Health Economics, 35, 123–131. 10.1016/j.jhealeco.2014.01.008 24662888 PMC4051992

[hec4907-bib-0042] Van Leeuwen, K. M. , Malley, J. , Bosmans, J. E. , Jansen, A. P. , Ostelo, R. W. , van der Horst, H. E. , & Netten, A. (2014). What can local authorities do to improve the social care‐related quality of life of older adults living at home? Evidence from the adult social care survey. Health & Place, 29, 104–113. 10.1016/j.healthplace.2014.06.004 25024121

[hec4907-bib-0043] Walsh, B. , Lyons, S. , Smith, S. , Wren, M. A. , Eighan, J. , & Morgenroth, E. (2020). Does formal home care reduce inpatient length of stay? Health Economics, 29(12), 1620–1636. 10.1002/hec.4158 32924255

[hec4907-bib-0044] Watkins, J. , Wulaningsih, W. , Da Zhou, C. , Marshall, D. C. , Sylianteng, G. D. , Rosa, P. G. D. , Miguel, V. A. , Raine, R. , King, L. P. , & Maruthappu, M. (2017). Effects of health and social care spending constraints on mortality in England: A time trend analysis. BMJ Open, 7(11), e017722. 10.1136/bmjopen-2017-017722 PMC5719267 29141897

[hec4907-bib-0045] Wooldridge, J. M. (2015). Control function methods in applied econometrics. Journal of Human Resources, 50(2), 420–445. 10.3368/jhr.50.2.420

[hec4907-bib-0046] Yogo, M. , & Stock, J. H. (2002). Testing for weak instruments in linear IV regression. National Bureau of Economic Research.

