# Supplementary material for: Unpacking the care‐related quality of life effect of England's publicly funded adult social care. A panel data analysis
Source: Health Econ. 2024 Oct 5;34(2):246–66. doi: 10.1002/hec.4907 (PMC11700955; doi:10.1002/hec.4907)
Supplement: Supplementary file 1 — Supporting Information S1 [file HEC-34-246-s001.docx]

Unpacking the Care-Related Quality of Life Effect of England's Publicly Funded Adult Social Care. A Panel Data Analysis

# Appendix

# Controlling for local authority fixed effects

Section 3.1 (The CRQoL effect of ASC expenditure and its channels) introduces regression (2) which estimates the effect of ASC expenditure on user CRQoL by controlling for time-invariant unobserved heterogeneity across local authorities (LAs) using LA fixed effects as follows:

$CRQoL_{ijt}=\alpha_{2}+\beta_{2}exp_{jt}+\theta_{2}Z_{ijt}+\pi_{2j}+\tau_{2t}+\varepsilon_{2ijkt}$, (2)

where CRQoL*_ijt_* is the Care-Related Quality of Life (CRQoL) score of user *i* (=1,…,*I*) in LA *j* (=1,…,*J*) at time *t* (=2014/15,…,2019/20), *α_2_* is the intercept, *exp_jt_* is publicly-funded ASC expenditure per long-term support (LTS) user, and *users_jt_* is the proportion of users; *Z_ijt_* is a vector of time-varying controls, *π_2j_* captures LA fixed effects, *τ_2t_* captures time fixed effects, and *ε_2ijt_* is the error term.

Controlling for LA fixed effects helps reduce the endogeneity of ASC expenditure per user. However, some endogeneity may remain due to the potential reverse causality and unobserved time-varying variables determining CRQoL and expenditure. As an example of these variables, users’ severity of illness may worsen over time determining the inability to carry out certain daily tasks which may lead to a CRQoL loss and the provision of more intensive ASC. To address this endogeneity, we use an instrument capturing the time-variability in the amount of missing council tax revenues driven by historical choices on the council tax charge during the freeze grant scheme period.

Council tax revenues are a major source of local funding that is mostly spent on ASC services (Local Government Association, 2017). They are determined by two components: the tax base and the tax charge. The tax base is a weighted count of domestic properties that can be argued to reflect historical features across LAs and that varies little over time. On the other hand, the tax charge (i.e., the amount paid by the occupiers of each domestic property) has been varying over time for multiple reasons including national policies. The council tax freeze grant scheme is one of these policies, started in 2011/12 and ended in 2015/16. During this period, LAs choosing to join this scheme received a freeze grant equivalent to a certain increase in the council tax charge (e.g. 2.5% in 2011/12) if they refrained from increasing their council tax charge. In addition, during the same years of the freeze grant scheme, LAs’ capacity to raise council tax revenues was restricted by the capping policy. LAs could increase the charge up to a maximum percentage of the charge, the cap, without running a local referendum that could reject increases above the cap. All LAs took the freeze grant in 2011/12 of an amount equivalent to an increase in the charge by 2.5%, although they could have instead increased the council tax charge up to a cap of 3.5%. However, the pattern of choices on the freeze grant varied across LAs subsequently. At the end of the freeze grant scheme, in 2016/17, the capping policy on the council tax charge increase continued which meant that LAs’ future capacity to raise council tax revenues remained constrained. Therefore, after the end of the scheme, LAs that did not increase the council tax charge and took the freeze grant became unable to fully offset this lack of increases that could have been made to past charges. Choosing not to cumulate charge increases in the past implies that the percentage charge increase today is applied to a lower charge. This generates a substantial amount of missing council tax revenues that tend to increase every year.^[[1]](#footnote-1)^ Therefore, we argue that historic decisions on council tax charges determining the participation of an LA in a ‘freeze grant scheme’ and the ‘capping policy’ are the key drivers of the contemporaneous time-variability in missing council tax revenues. This is likely to explain the time-variability in ASC expenditure (relevance condition) and, since unanticipated, it is also likely to be independent of time-variability in ASC outcomes and needs (exogeneity condition).

We calculate the amount of missing council tax revenues for each LA taking account of the pattern of choices on the council tax charge since 2011/12 (when the freeze grant was introduced). For example, consider the missing council tax revenues in 2014/15 for those LAs that chose to take the freeze grant every year since 2011/12 by keeping the council tax charge unchanged or by reducing it. For these LAs, first, we calculate the amount of actual council tax revenues in 2014/15 by multiplying their council tax charge in 2014/15 (which is expected to be at the same or lower level than their charge in 2010/11) by their tax base in 2014/15, and by adding the amount of the freeze grant that they received in 2011/12, 2013/14 and 2014/15. We then subtract this quantity from the hypothetical amount of revenues that these LAs could have made in 2014/15 had they chosen to increase the council tax charge by the cap every year since 2011/12. The missing council tax revenues calculated in this way are used to obtain our two instruments: missing council tax revenues per user and its square.

In addition, regression (2) excludes the proportion of users as an additional control variable since it is almost time-invariant and, therefore, mostly captured through the LA fixed effects. Table 2 shows that the overall standard deviation (0.48%) of this variable is almost totally explained by variability across LAs (between-group standard deviation, 0.46%) rather than variability over time (within-group standard deviation, 0.17%). Moreover, conditional on the time-varying covariates and fixed effects the remaining within-group standard deviation in the proportion of users is reduced to less than one-fifth of the overall standard deviation (0.09%) – a negligible amount. Finally, we estimate regression (2) by 2SLS, weighing observations by their survey weights, and clustering standard errors within LAs and strata.

# Results from the LA fixed effects analysis of channels and other outcomes

As discussed in Section 3.1 (The CRQoL effect of ASC expenditure and its channels), regression (2) is likely to estimate a short-run effect by analysing contemporaneous time-variability across LAs (e.g., Kuh, 1959, Houthakker, 1965). As discussed in Section 4 (Results), estimates from (2) indicate that a £1,000 increase in ASC expenditure per user increases on average user CRQoL by 0.002. Table A6 shows that this CRQoL gain is driven by the gains for users aged 65 or older with no learning disability receiving care in the community. In other words, these users are the only ones likely to obtain a short-term CRQoL benefit. As still shown in Table A6, this short-term benefit occurs because ASC expenditure statistically significantly (at the 5% level) improves the safety, social participation, occupation, control over daily life and dignity (CRQoL) domains. This occurs because community care users tend to receive support from professional carers who can also provide users with a chance for social interaction and help with housework in their homes. Moreover, these users may receive home adaptations and equipment to improve safety.

# Appendix Tables

Table A1 – Data sources.

| Variable | Original unit | Unit of analysis | Financial year | Source | Last access |
| --- | --- | --- | --- | --- | --- |
| [Long-term support user characteristics and outcomes](https://digital.nhs.uk/data-and-information/publications/statistical/personal-social-services-adult-social-care-survey) | Individual | Individual | 2014/15-2019/20 | NHS Digital: Personal Social Services Adult Social Care Survey | 31/07/2024 |
| [Publicly-funded Adult Social Care expenditure and number of users](https://digital.nhs.uk/data-and-information/publications/statistical/adult-social-care-activity-and-finance-report/) | Local authority | Local authority | 2014/15-2019/20 | NHS Digital: Adult Social Care Activity and Finance Report | 31/07/2024 |
| [Population characteristics](https://census.ukdataservice.ac.uk/get-data/aggregate-data) | LSOA | Local authority | 2011 | 2011 Census | 31/07/2024 |
| [Index of multiple deprivation](http://opendatacommunities.org/resource?uri=http%3A%2F%2Fopendatacommunities.org%2Fdata%2Fsocietal-wellbeing%2Fimd%2Findices) | LSOA | Local authority | 2015 | Department for Levelling Up, Housing and Communities | 31/07/2024 |
| [Council tax base](https://www.gov.uk/government/collections/council-tax-statistics) | Local authority | Local authority | 2011/2-2019/20 | Government | 31/07/2024 |
| [Business rate tax base](https://www.gov.uk/government/collections/non-domestic-rating-stock-of-properties-collection) | Local authority | Local authority | 2014/15-2019/20 | Government | 31/07/2024 |
| [Freeze Grant Scheme](https://www.gov.uk/government/collections/council-tax-freeze-scheme) | Local authority | Local authority | 2011/12-2015/16 | Department for Levelling Up, Housing and Communities | 31/07/2024 |

Table A2 – Descriptive statistics on user-level variables in 2015/16 and 2016/17.

| Variable | | | 2015/16 | | |  | 2016/17 | | |
| --- | --- | --- | --- | --- | --- | --- | --- | --- | --- |
|  |  |  | Obs | Mean | SD |  | Obs | Mean | SD |
| CRQoL and its domains | CRQoL score | | 61,179 | 0.815 | 0.193 |  | 61,394 | 0.815 | 0.193 |
|  | CRQoL score for (a) users with a learning disability | | 14,777 | 0.912 | 0.112 |  | 15,650 | 0.910 | 0.115 |
|  | CRQoL score for (b) users with no learning disability aged between 18-64 | | 12,373 | 0.752 | 0.230 |  | 11,859 | 0.756 | 0.231 |
|  | CRQoL score for (c) users with no learning disability aged 65+ in residential or nursing care | | 10,311 | 0.855 | 0.173 |  | 10,267 | 0.851 | 0.169 |
|  | CRQoL score for (d) users with no learning disability aged 65+ using community-based care | | 23,718 | 0.775 | 0.195 |  | 23,618 | 0.775 | 0.196 |
|  | Control over daily life score | | 69,101 | 0.109 | 0.052 |  | 68,576 | 0.111 | 0.051 |
|  | Personal cleanliness and comfort score | | 69,115 | 0.110 | 0.030 |  | 68,771 | 0.110 | 0.030 |
|  | Food and drink score | | 68,533 | 0.107 | 0.028 |  | 68,050 | 0.107 | 0.028 |
|  | Accommodation cleanliness and comfort score | | 68,636 | 0.108 | 0.021 |  | 68,521 | 0.108 | 0.021 |
|  | Safety score | | 68,957 | 0.091 | 0.045 |  | 68,442 | 0.092 | 0.045 |
|  | Social participation and involvement score | | 68,504 | 0.091 | 0.036 |  | 68,269 | 0.091 | 0.036 |
|  | Occupation (time spent in enjoyable activities) score | | 67,760 | 0.103 | 0.049 |  | 67,532 | 0.102 | 0.049 |
|  | Dignity score | | 66,844 | 0.091 | 0.034 |  | 66,718 | 0.091 | 0.034 |
| ASC performance | Improved quality of life (by support) | ASC services helped | 67,933 | 7.7 | 26.6 |  | 67,317 | 7.4 | 26.1 |
|  |  | ASC services did not help | 67,933 | 92.3 | 26.6 |  | 67,317 | 92.6 | 26.1 |
|  | User satisfaction with support | Very | 69,092 | 63.8 | 48.1 |  | 68,747 | 64.3 | 47.9 |
|  |  | Quite | 69,092 | 26.6 | 44.2 |  | 68,747 | 25.9 | 43.8 |
|  |  | Neither satisfied nor dissatisfied | 69,092 | 6.1 | 23.8 |  | 68,747 | 6.0 | 23.8 |
|  |  | Quite dissatisfied | 69,092 | 2.1 | 14.3 |  | 68,747 | 2.1 | 14.2 |
|  |  | Vey dissatisfied | 69,092 | 1.5 | 12.3 |  | 68,747 | 1.7 | 12.9 |
|  | User experience with finding information and advice | Very easy | 66,579 | 19.9 | 39.9 |  | 66,403 | 20.1 | 40.1 |
|  |  | Fairly easy | 66,579 | 33.7 | 47.3 |  | 66,403 | 33.5 | 47.2 |
|  |  | Fairly difficult | 66,579 | 13.1 | 33.8 |  | 66,403 | 13.3 | 33.9 |
|  |  | Very difficult | 66,579 | 6.6 | 24.7 |  | 66,403 | 6.7 | 25.1 |
|  |  | Never tried | 66,579 | 26.8 | 44.3 |  | 66,403 | 26.4 | 44.1 |
|  | Getting in and out of bed | I can do it easily | 68,611 | 52.1 | 50.0 |  | 68,175 | 51.9 | 50.0 |
|  |  | I have difficulty doing it | 68,611 | 23.7 | 42.5 |  | 68,175 | 23.6 | 42.5 |
|  |  | I cannot do it | 68,611 | 24.3 | 42.9 |  | 68,175 | 24.6 | 43.0 |
| Activities of daily living by themselves | Washing face and hands | I can do it easily | 68,806 | 68.3 | 46.5 |  | 68,418 | 67.0 | 47.0 |
|  |  | I have difficulty doing it | 68,806 | 16.5 | 37.1 |  | 68,418 | 17.2 | 37.7 |
|  |  | I cannot do it | 68,806 | 15.2 | 35.9 |  | 68,418 | 15.8 | 36.5 |
|  | Bathing and showering | I can do it easily | 68,636 | 26.9 | 44.3 |  | 68,394 | 27.2 | 44.5 |
|  |  | I have difficulty doing it | 68,636 | 26.4 | 44.1 |  | 68,394 | 26.0 | 43.9 |
|  |  | I cannot do it | 68,636 | 46.7 | 49.9 |  | 68,394 | 46.8 | 49.9 |
|  | Using the toilet | I can do it easily | 68,478 | 57.9 | 49.4 |  | 68,192 | 56.8 | 49.5 |
|  |  | I have difficulty doing it | 68,478 | 18.3 | 38.7 |  | 68,192 | 19.0 | 39.3 |
|  |  | I cannot do it | 68,478 | 23.8 | 42.6 |  | 68,192 | 24.2 | 42.8 |
|  | Dressing | I can do it easily | 68,551 | 38.6 | 48.7 |  | 68,294 | 38.3 | 48.6 |
|  |  | I have difficulty doing it | 68,551 | 26.1 | 43.9 |  | 68,294 | 26.1 | 43.9 |
|  |  | I cannot do it | 68,551 | 35.2 | 47.8 |  | 68,294 | 35.5 | 47.9 |
|  | Feeding | I can do it easily | 68,607 | 76.5 | 42.4 |  | 68,174 | 75.3 | 43.1 |
|  |  | I have difficulty doing it | 68,607 | 16.0 | 36.6 |  | 68,174 | 16.5 | 37.1 |
|  |  | I cannot do it | 68,607 | 7.5 | 26.3 |  | 68,174 | 8.2 | 27.4 |
|  | Getting around indoors | I can do it easily | 68,451 | 49.7 | 50.0 |  | 68,088 | 49.7 | 50.0 |
|  |  | I have difficulty doing it | 68,451 | 27.9 | 44.8 |  | 68,088 | 27.5 | 44.7 |
|  |  | I cannot do it | 68,451 | 22.5 | 41.7 |  | 68,088 | 22.8 | 42.0 |
|  | Doing some paperwork | I can do it easily | 68,172 | 18.9 | 39.1 |  | 67,832 | 18.0 | 38.4 |
|  |  | I have difficulty doing it | 68,172 | 15.6 | 36.3 |  | 67,832 | 15.5 | 36.2 |
|  |  | I cannot do it | 68,172 | 65.5 | 47.6 |  | 67,832 | 66.5 | 47.2 |
| Surroundings and needs | Home design | Meets my needs very well | 67,995 | 55.4 | 49.7 |  | 67,898 | 55.1 | 49.7 |
|  |  | Meets most of my needs | 67,995 | 31.2 | 46.3 |  | 67,898 | 31.4 | 46.4 |
|  |  | Meets some of my needs | 67,995 | 11.0 | 31.2 |  | 67,898 | 10.9 | 31.2 |
|  |  | Totally inappropriate | 67,995 | 2.5 | 15.7 |  | 67,898 | 2.7 | 16.1 |
|  | Getting around outdoors | I can get to all places | 67,176 | 28.3 | 45.1 |  | 67,041 | 28.3 | 45.1 |
|  |  | At times, I find it difficult | 67,176 | 22.3 | 41.6 |  | 67,041 | 22.2 | 41.6 |
|  |  | I am unable to get to all places | 67,176 | 21.8 | 41.3 |  | 67,041 | 21.4 | 41.0 |
|  |  | I do not leave my home | 67,176 | 27.6 | 44.7 |  | 67,041 | 28.1 | 44.9 |
| Other care | Unpaid informal care | Receiving this form of care | 67,362 | 19.0 | 39.3 |  | 67,137 | 19.0 | 39.2 |
|  | Unpaid informal care | Not receiving this form of care | 67,362 | 81.0 | 39.3 |  | 67,137 | 81.0 | 39.2 |
|  | Privately-funded care | Receiving this form of care | 65,922 | 62.7 | 48.4 |  | 65,757 | 63.1 | 48.3 |
|  | Privately-funded care | Not receiving this form of care | 65,922 | 37.3 | 48.4 |  | 65,757 | 36.9 | 48.3 |
| Health | General health | Very good | 68,857 | 13.9 | 34.6 |  | 68,640 | 13.9 | 34.6 |
|  |  | Good | 68,857 | 26.9 | 44.3 |  | 68,640 | 26.6 | 44.2 |
|  |  | Fair | 68,857 | 41.6 | 49.3 |  | 68,640 | 41.1 | 49.2 |
|  |  | Bad | 68,857 | 13.1 | 33.7 |  | 68,640 | 13.5 | 34.1 |
|  |  | Very bad | 68,857 | 4.6 | 20.9 |  | 68,640 | 5.0 | 21.7 |
|  | EQ-5D: pain and discomfort | No | 68,206 | 34.1 | 47.4 |  | 68,018 | 34.8 | 47.6 |
|  |  | Moderate | 68,206 | 52.5 | 49.9 |  | 68,018 | 51.7 | 50.0 |
|  |  | Extreme | 68,206 | 13.4 | 34.1 |  | 68,018 | 13.6 | 34.2 |
|  | EQ-5D: anxiety and depression | No | 67,503 | 47.3 | 49.9 |  | 67,298 | 46.3 | 49.9 |
|  |  | Moderate | 67,503 | 45.1 | 49.8 |  | 67,298 | 45.6 | 49.8 |
|  |  | Extreme | 67,503 | 7.7 | 26.6 |  | 67,298 | 8.2 | 27.4 |
| User characteristics | Female | | 198,038 | 61.6 | 2.8 |  | 196,465 | 61.1 | 3.2 |
|  | Aged 65 or older | | 198,038 | 67.8 | 4.9 |  | 196,465 | 66.9 | 4.9 |
|  | Non-white ethnicity | | 198,038 | 8.7 | 13.3 |  | 196,465 | 9.2 | 13.4 |
|  | Questionnaire in English | | 198,038 | 99.9 | 1.2 |  | 196,465 | 99.9 | 1.0 |
|  | Help with the questionnaire | No help | 198,038 | 19.7 | 4.4 |  | 196,465 | 19.6 | 4.2 |
|  |  | Read by someone else | 198,038 | 47.6 | 4.6 |  | 196,465 | 46.1 | 5.0 |
|  |  | Translated by someone else | 198,038 | 19.0 | 4.1 |  | 196,465 | 19.6 | 4.2 |
|  |  | Written by someone else | 198,038 | 40.0 | 5.3 |  | 196,465 | 39.5 | 4.7 |
|  |  | Talked through with someone else | 198,038 | 28.8 | 3.7 |  | 196,465 | 29.7 | 3.7 |
|  |  | Answered by someone else without asking | 198,038 | 9.6 | 2.6 |  | 196,465 | 10.3 | 3.4 |
|  | Easy-read version of the questionnaire | | 198,038 | 83.5 | 2.8 |  | 196,465 | 82.6 | 4.2 |
|  | Primary support reason | Physical support | 198,038 | 61.1 | 7.5 |  | 196,465 | 60.3 | 7.3 |
|  |  | Sensory support | 198,038 | 1.4 | 1.1 |  | 196,465 | 1.5 | 1.1 |
|  |  | Support with memory and cognition | 198,038 | 8.5 | 5.8 |  | 196,465 | 8.5 | 5.6 |
|  |  | Learning disability support | 198,038 | 16.5 | 2.8 |  | 196,465 | 16.8 | 2.9 |
|  |  | Mental health support | 198,038 | 10.8 | 7.0 |  | 196,465 | 10.7 | 7.0 |
|  |  | Social support | 198,038 | 1.7 | 1.9 |  | 196,465 | 2.2 | 2.0 |
| Obs=observations, SD=standard deviation, CRQoL=Care-Related Quality of Life, ASC=Adult Social Care | | | | | | | | | |
| Mean and standard deviation are weighted by the survey weight. | | | | | | | | | |

Table A3 – Descriptive statistics on user-level variables in 2017/18 and 2018/19.

| Variable | | | 2017/18 | | |  | 2018/19 | | |
| --- | --- | --- | --- | --- | --- | --- | --- | --- | --- |
|  |  |  | Obs | Mean | SD |  | Obs | Mean | SD |
| CRQoL and its domains | CRQoL score | | 55,288 | 0.818 | 0.193 |  | 58,221 | 0.817 | 0.194 |
|  | CRQoL score for (a) users with a learning disability | | 15,309 | 0.912 | 0.111 |  | 16,044 | 0.909 | 0.117 |
|  | CRQoL score for (b) users with no learning disability aged between 18-64 | | 10,479 | 0.759 | 0.231 |  | 11,133 | 0.758 | 0.231 |
|  | CRQoL score for (c) users with no learning disability aged 65+ in residential or nursing care | | 9,044 | 0.851 | 0.173 |  | 9,641 | 0.850 | 0.173 |
|  | CRQoL score for (d) users with no learning disability aged 65+ using community-based care | | 20,456 | 0.774 | 0.198 |  | 21,403 | 0.773 | 0.199 |
|  | Control over daily life score | | 61,518 | 0.112 | 0.051 |  | 65,034 | 0.111 | 0.051 |
|  | Personal cleanliness and comfort score | | 61,669 | 0.111 | 0.030 |  | 65,247 | 0.110 | 0.031 |
|  | Food and drink score | | 61,134 | 0.107 | 0.028 |  | 64,581 | 0.107 | 0.029 |
|  | Accommodation cleanliness and comfort score | | 61,496 | 0.108 | 0.021 |  | 64,999 | 0.108 | 0.021 |
|  | Safety score | | 61,495 | 0.092 | 0.045 |  | 65,001 | 0.092 | 0.045 |
|  | Social participation and involvement score | | 61,235 | 0.091 | 0.036 |  | 64,808 | 0.091 | 0.036 |
|  | Occupation (time spent in enjoyable activities) score | | 60,697 | 0.104 | 0.049 |  | 64,090 | 0.104 | 0.049 |
|  | Dignity score | | 60,023 | 0.091 | 0.034 |  | 63,256 | 0.091 | 0.035 |
| ASC performance | Improved quality of life (by support) | ASC services helped | 60,558 | 7.5 | 26.3 |  | 63,745 | 7.8 | 26.8 |
|  |  | ASC services did not help | 60,558 | 92.5 | 26.3 |  | 63,745 | 92.2 | 26.8 |
|  | User satisfaction with support | Very | 61,698 | 64.9 | 47.7 |  | 65,228 | 64.0 | 48.0 |
|  |  | Quite | 61,698 | 25.3 | 43.4 |  | 65,228 | 25.5 | 43.6 |
|  |  | Neither satisfied nor dissatisfied | 61,698 | 5.8 | 23.4 |  | 65,228 | 6.2 | 24.1 |
|  |  | Quite dissatisfied | 61,698 | 2.2 | 14.5 |  | 65,228 | 2.4 | 15.3 |
|  |  | Vey dissatisfied | 61,698 | 1.8 | 13.5 |  | 65,228 | 1.9 | 13.7 |
|  | User experience with finding information and advice | Very easy | 59,669 | 20.5 | 40.3 |  | 62,800 | 43.4 | 49.6 |
|  |  | Fairly easy | 59,669 | 33.5 | 47.2 |  | 62,800 | 16.8 | 37.4 |
|  |  | Fairly difficult | 59,669 | 12.9 | 33.5 |  | 62,800 | 22.0 | 41.4 |
|  |  | Very difficult | 59,669 | 7.0 | 25.6 |  | 62,800 | 11.0 | 31.3 |
|  |  | Never tried | 59,669 | 26.1 | 43.9 |  | 62,800 | 6.7 | 25.0 |
|  | Getting in and out of bed | I can do it easily | 61,235 | 53.4 | 49.9 |  | 64,522 | 53.8 | 49.9 |
|  |  | I have difficulty doing it | 61,235 | 22.3 | 41.6 |  | 64,522 | 22.1 | 41.5 |
|  |  | I cannot do it | 61,235 | 24.3 | 42.9 |  | 64,522 | 24.1 | 42.8 |
| Activities of daily living by themselves | Washing face and hands | I can do it easily | 61,223 | 67.3 | 46.9 |  | 64,805 | 68.1 | 46.6 |
|  |  | I have difficulty doing it | 61,223 | 16.6 | 37.2 |  | 64,805 | 16.3 | 36.9 |
|  |  | I cannot do it | 61,223 | 16.1 | 36.8 |  | 64,805 | 15.7 | 36.3 |
|  | Bathing and showering | I can do it easily | 61,125 | 28.6 | 45.2 |  | 64,687 | 28.8 | 45.3 |
|  |  | I have difficulty doing it | 61,125 | 25.6 | 43.6 |  | 64,687 | 26.2 | 44.0 |
|  |  | I cannot do it | 61,125 | 45.9 | 49.8 |  | 64,687 | 45.0 | 49.7 |
|  | Using the toilet | I can do it easily | 60,997 | 57.6 | 49.4 |  | 64,485 | 57.9 | 49.4 |
|  |  | I have difficulty doing it | 60,997 | 18.4 | 38.7 |  | 64,485 | 18.1 | 38.5 |
|  |  | I cannot do it | 60,997 | 24.0 | 42.7 |  | 64,485 | 24.1 | 42.8 |
|  | Dressing | I can do it easily | 61,036 | 39.3 | 48.8 |  | 64,591 | 39.8 | 48.9 |
|  |  | I have difficulty doing it | 61,036 | 25.8 | 43.8 |  | 64,591 | 25.7 | 43.7 |
|  |  | I cannot do it | 61,036 | 34.9 | 47.7 |  | 64,591 | 34.5 | 47.5 |
|  | Feeding | I can do it easily | 61,171 | 75.5 | 43.0 |  | 64,580 | 75.5 | 43.0 |
|  |  | I have difficulty doing it | 61,171 | 16.3 | 36.9 |  | 64,580 | 16.5 | 37.1 |
|  |  | I cannot do it | 61,171 | 8.2 | 27.5 |  | 64,580 | 8.1 | 27.2 |
|  | Getting around indoors | I can do it easily | 61,101 | 51.0 | 50.0 |  | 64,309 | 51.1 | 50.0 |
|  |  | I have difficulty doing it | 61,101 | 26.6 | 44.2 |  | 64,309 | 26.2 | 44.0 |
|  |  | I cannot do it | 61,101 | 22.4 | 41.7 |  | 64,309 | 22.7 | 41.9 |
|  | Doing some paperwork | I can do it easily | 60,799 | 17.7 | 38.2 |  | 64,201 | 17.7 | 38.2 |
|  |  | I have difficulty doing it | 60,799 | 15.4 | 36.1 |  | 64,201 | 15.1 | 35.8 |
|  |  | I cannot do it | 60,799 | 66.9 | 47.0 |  | 64,201 | 67.2 | 46.9 |
| Surroundings and needs | Home design | Meets my needs very well | 60,953 | 55.2 | 49.7 |  | 64,342 | 55.0 | 49.8 |
|  |  | Meets most of my needs | 60,953 | 31.3 | 46.4 |  | 64,342 | 31.2 | 46.3 |
|  |  | Meets some of my needs | 60,953 | 10.9 | 31.1 |  | 64,342 | 11.0 | 31.3 |
|  |  | Totally inappropriate | 60,953 | 2.6 | 16.0 |  | 64,342 | 2.8 | 16.6 |
|  | Getting around outdoors | I can get to all places | 60,072 | 29.4 | 45.6 |  | 63,427 | 29.3 | 45.5 |
|  |  | At times, I find it difficult | 60,072 | 22.5 | 41.8 |  | 63,427 | 22.9 | 42.0 |
|  |  | I am unable to get to all places | 60,072 | 21.1 | 40.8 |  | 63,427 | 21.0 | 40.7 |
|  |  | I do not leave my home | 60,072 | 27.0 | 44.4 |  | 63,427 | 26.8 | 44.3 |
| Other care | Unpaid informal care | Receiving this form of care | 60,029 | 19.1 | 39.3 |  | 63,003 | 19.2 | 39.4 |
|  | Unpaid informal care | Not receiving this form of care | 60,029 | 80.9 | 39.3 |  | 63,003 | 80.8 | 39.4 |
|  | Privately-funded care | Receiving this form of care | 58,762 | 61.7 | 48.6 |  | 61,975 | 61.2 | 48.7 |
|  | Privately-funded care | Not receiving this form of care | 58,762 | 38.3 | 48.6 |  | 61,975 | 38.8 | 48.7 |
| Health | General health | Very good | 61,545 | 15.1 | 35.8 |  | 65,007 | 14.4 | 35.1 |
|  |  | Good | 61,545 | 27.0 | 44.4 |  | 65,007 | 27.5 | 44.6 |
|  |  | Fair | 61,545 | 40.3 | 49.1 |  | 65,007 | 40.5 | 49.1 |
|  |  | Bad | 61,545 | 12.9 | 33.6 |  | 65,007 | 13.2 | 33.8 |
|  |  | Very bad | 61,545 | 4.6 | 20.9 |  | 65,007 | 4.5 | 20.7 |
|  | EQ-5D: pain and discomfort | No | 60,968 | 36.4 | 48.1 |  | 64,395 | 35.7 | 47.9 |
|  |  | Moderate | 60,968 | 50.6 | 50.0 |  | 64,395 | 50.8 | 50.0 |
|  |  | Extreme | 60,968 | 13.1 | 33.7 |  | 64,395 | 13.5 | 34.2 |
|  | EQ-5D: anxiety and depression | No | 60,443 | 45.9 | 49.8 |  | 63,678 | 49.6 | 50.0 |
|  |  | Moderate | 60,443 | 46.1 | 49.8 |  | 63,678 | 42.1 | 49.4 |
|  |  | Extreme | 60,443 | 8.0 | 27.2 |  | 63,678 | 8.3 | 27.5 |
| User characteristics | Female | | 195,391 | 60.4 | 2.6 |  | 209,300 | 59.9 | 3.0 |
|  | Aged 65 or older | | 195,391 | 66.3 | 4.9 |  | 209,300 | 65.2 | 4.7 |
|  | Non-white ethnicity | | 194,441 | 9.8 | 13.7 |  | 209,300 | 9.8 | 14.3 |
|  | Questionnaire in English | | 195,391 | 99.9 | 1.2 |  | 209,300 | 99.8 | 1.6 |
|  | Help with the questionnaire | No help | 195,391 | 19.6 | 4.4 |  | 209,300 | 20.0 | 3.9 |
|  |  | Read by someone else | 195,391 | 47.1 | 5.7 |  | 209,300 | 47.0 | 5.1 |
|  |  | Translated by someone else | 195,391 | 21.1 | 4.3 |  | 209,300 | 20.7 | 6.1 |
|  |  | Written by someone else | 195,391 | 39.5 | 4.8 |  | 209,300 | 37.7 | 4.5 |
|  |  | Talked through with someone else | 195,391 | 28.6 | 3.9 |  | 209,300 | 29.2 | 3.6 |
|  |  | Answered by someone else without asking | 195,391 | 10.3 | 3.1 |  | 209,300 | 10.5 | 2.4 |
|  | Easy-read version of the questionnaire | | 195,391 | 82.7 | 5.6 |  | 209,300 | 82.6 | 5.4 |
|  | Primary support reason | Physical support | 195,391 | 59.7 | 7.3 |  | 209,300 | 58.8 | 6.3 |
|  |  | Sensory support | 195,391 | 1.5 | 1.0 |  | 209,300 | 1.4 | 0.9 |
|  |  | Support with memory and cognition | 195,391 | 8.5 | 5.1 |  | 209,300 | 8.7 | 4.9 |
|  |  | Learning disability support | 195,391 | 17.2 | 3.2 |  | 209,300 | 17.8 | 3.1 |
|  |  | Mental health support | 195,391 | 10.9 | 6.8 |  | 209,300 | 11.1 | 6.3 |
|  |  | Social support | 195,391 | 2.2 | 2.0 |  | 209,300 | 2.4 | 1.9 |
| Obs=observations, SD=standard deviation, CRQoL=Care-Related Quality of Life, ASC=Adult Social Care | | | | | | | | | |
| Mean and standard deviation are weighted by the survey weight. | | | | | | | | | |

Table A4a– Full results from regression (1) estimated by OLS and 2SLS

| Variable | **Regression (1)** | | | | |
| --- | --- | --- | --- | --- | --- |
|  | OLS | 2SLS | | | |
|  |  | First stage | | | Second stage |
|  |  | ASC expenditure per user | ASC expenditure per user, squared | Prop. of users |  |
| ASC expenditure per LTS user at mean level | 0.005** |  |  |  | 0.031*** |
|  | (0.001 - 0.009) |  |  |  | (0.010 - 0.051) |
| ASC expenditure per LTS user, squared | -0.00007** |  |  |  | -0.0003*** |
|  | (-0.000 - -0.000) |  |  |  | (-0.001 - -0.000) |
| Prop. of LTS users | 0.002 |  |  |  | 0.096** |
| Council tax base per user at zero level | (-0.012 - 0.016) | 1.293*** | 44.529*** | -0.188*** | (0.018 - 0.174) |
|  |  | [1.125,1.462] | [33.814,55.244] | [-0.201,-0.176] |  |
| Council tax base per user, squared |  | -0.010*** | -0.169 | 0.002*** |  |
|  |  | [-0.014,-0.007] | [-0.386,0.048] | [0.002,0.002] |  |
| County authority |  | 0.203 | -0.373 | -0.128*** |  |
|  |  | [-1.186,1.592] | [-73.530,72.783] | [-0.182,-0.074] |  |
| Metropolitan authority |  | 0.791 | 47.606 | -0.171*** |  |
|  |  | [-0.339,1.921] | [-11.343,106.554] | [-0.222,-0.120] |  |
| Unitary authority |  | 1.265** | 61.214* | -0.129*** |  |
|  |  | [0.058,2.472] | [-2.110,124.537] | [-0.180,-0.078] |  |
| Missing council tax revenues per user |  |  |  |  |  |
|  |  |  |  |  |  |
| Missing council tax revenues per user, squared |  |  |  |  |  |
|  |  |  |  |  |  |
| Female | -0.003** | 0.004 | 0.203 | -0.001 | -0.003** |
|  | (-0.006 - -0.001) | [-0.019,0.027] | [-0.946,1.353] | [-0.002,0.001] | (-0.006 - -0.001) |
| Aged 65 or older | 0.039*** | 0.002 | 0.279 | -0.001 | 0.040*** |
|  | (0.034 - 0.045) | [-0.267,0.271] | [-12.953,13.512] | [-0.014,0.011] | (0.033 - 0.046) |
| White ethnicity | 0.030*** | 0.05 | 2.438 | 0.003 | 0.029*** |
|  | (0.025 - 0.034) | [-0.044,0.144] | [-2.181,7.057] | [-0.002,0.007] | (0.024 - 0.033) |
| Questionnaire in English | 0.057*** | -0.11 | -1.526 | -0.016 | 0.061*** |
|  | (0.028 - 0.085) | [-0.939,0.719] | [-45.312,42.260] | [-0.041,0.009] | (0.031 - 0.090) |
| No help with the questionnaire | 0.017*** | -0.037 | -1.861 | 0.0002 | 0.018*** |
|  | (0.012 - 0.022) | [-0.093,0.019] | [-4.648,0.926] | [-0.002,0.003] | (0.012 - 0.023) |
| The questionnaire was read by someone else | 0.035*** | -0.023 | -1.315 | -0.0003 | 0.035*** |
|  | (0.032 - 0.038) | [-0.061,0.014] | [-3.190,0.559] | [-0.002,0.001] | (0.032 - 0.038) |
| The questionnaire’s questions were translated by someone else | 0.003** | 0.037* | 2.198* | 0.001 | 0.003* |
|  | (0.000 - 0.006) | [-0.006,0.081] | [-0.009,4.406] | [-0.000,0.003] | (-0.000 - 0.005) |
| The questionnaire’s answers were written by someone else | -0.015*** | -0.050*** | -2.409*** | 0.00002 | -0.015*** |
|  | (-0.018 - -0.013) | [-0.079,-0.020] | [-3.895,-0.924] | [-0.001,0.001] | (-0.017 - -0.013) |
| The questionnaire’s questions were talked through with someone else | -0.017*** | -0.004 | -0.326 | -0.001** | -0.017*** |
|  | (-0.019 - -0.015) | [-0.029,0.021] | [-1.551,0.898] | [-0.002,-0.000] | (-0.019 - -0.014) |
| The questionnaire’s questions were answered by someone else without asking | -0.066*** | -0.03 | -1.144 | -0.001 | -0.066*** |
|  | (-0.071 - -0.061) | [-0.078,0.018] | [-3.598,1.310] | [-0.003,0.001] | (-0.070 - -0.061) |
| Easy-read version of the questionnaire | 0.044*** | -0.136 | -3.671 | 0.011 | 0.045*** |
|  | (0.027 - 0.061) | [-0.593,0.322] | [-26.360,19.019] | [-0.005,0.028] | (0.027 - 0.064) |
| Sensory support | -0.003 | 0.044 | 1.851 | -0.003 | -0.003 |
|  | (-0.011 - 0.006) | [-0.110,0.199] | [-6.308,10.011] | [-0.009,0.003] | (-0.012 - 0.006) |
| Support with memory and cognition | 0.031*** | -0.039 | -2.349 | -0.0002 | 0.031*** |
|  | (0.024 - 0.037) | [-0.183,0.104] | [-9.473,4.776] | [-0.007,0.006] | (0.024 - 0.037) |
| Learning disability support | 0.101*** | 0.174 | 5.667 | -0.015 | 0.100*** |
|  | (0.085 - 0.118) | [-0.360,0.708] | [-21.130,32.464] | [-0.035,0.006] | (0.082 - 0.119) |
| Mental health support | 0.013*** | -0.099* | -4.476 | 0.004 | 0.013*** |
|  | (0.006 - 0.019) | [-0.210,0.012] | [-9.927,0.976] | [-0.002,0.010] | (0.007 - 0.020) |
| Social support | 0.008* | 0.026 | 0.084 | -0.005 | 0.008* |
|  | (-0.001 - 0.017) | [-0.135,0.187] | [-7.819,7.987] | [-0.012,0.003] | (-0.001 - 0.017) |
| Prop. carers who are female | 0.0002 | -0.040** | -1.231 | 0.003*** | 0.001* |
|  | (-0.000 - 0.001) | [-0.074,-0.006] | [-2.893,0.431] | [0.002,0.004] | (-0.000 - 0.002) |
| Prop. carers aged 65 or older | -0.00006 | -0.041*** | -1.878*** | -0.002*** | 0.0004 |
|  | (-0.001 - 0.000) | [-0.066,-0.015] | [-3.173,-0.583] | [-0.003,-0.000] | (-0.000 - 0.001) |
| Prop. carers who are white | -0.00007 | 0.020*** | 1.041*** | 0.0002 | -0.0003** |
|  | (-0.000 - 0.000) | [0.012,0.029] | [0.623,1.459] | [-0.000,0.001] | (-0.001 - -0.000) |
| Prop. carers who are retired | 0.00004 | -0.003 | -0.536 | -0.002* | 0.0002 |
|  | (-0.001 - 0.001) | [-0.048,0.041] | [-2.842,1.769] | [-0.004,0.000] | (-0.001 - 0.001) |
| Prop. carers who are employed full-time | -0.001* | -0.035 | -3.032* | 0.001 | -0.001 |
|  | (-0.003 - 0.000) | [-0.106,0.035] | [-6.645,0.580] | [-0.002,0.004] | (-0.003 - 0.000) |
| Prop. carers who are employed part-time | 0.00007 | 0.104*** | 4.282** | -0.009*** | -0.0003 |
|  | (-0.001 - 0.001) | [0.039,0.169] | [1.024,7.540] | [-0.012,-0.006] | (-0.002 - 0.001) |
| Prop. carers who are self-employed full-time | 0.002* | -0.062 | -1.597 | -0.0003 | 0.003** |
|  | (-0.000 - 0.005) | [-0.176,0.051] | [-7.483,4.289] | [-0.006,0.006] | (0.000 - 0.006) |
| Prop. carers who are self-employed part-time | -0.001 | -0.033 | -1.293 | 0.006*** | -0.0002 |
|  | (-0.002 - 0.001) | [-0.122,0.056] | [-6.028,3.441] | [0.002,0.011] | (-0.002 - 0.002) |
| Prop. carers who are not in paid work | -0.001 | 0.094*** | 4.518*** | -0.005*** | -0.001* |
|  | (-0.001 - 0.000) | [0.046,0.143] | [2.083,6.953] | [-0.007,-0.003] | (-0.002 - 0.000) |
| Prop. carers who are doing voluntary work | -0.001 | -0.006 | 0.692 | -0.003 | -0.0002 |
|  | (-0.002 - 0.001) | [-0.094,0.081] | [-3.883,5.266] | [-0.006,0.001] | (-0.002 - 0.001) |
| Prop. carers who are doing other type of work | -0.001 | -0.075** | -4.281** | 0.001 | -0.0003 |
|  | (-0.002 - 0.001) | [-0.146,-0.004] | [-7.848,-0.714] | [-0.002,0.003] | (-0.002 - 0.002) |
| Prop. carers with physical impairment or disability | 0.001 | -0.004 | 0.327 | 0.00008 | 0.001 |
|  | (-0.000 - 0.002) | [-0.044,0.035] | [-1.708,2.361] | [-0.002,0.002] | (-0.000 - 0.002) |
| Prop. carers with sight or hearing loss | -0.000 | 0.097*** | 4.455*** | 0.001 | -0.001 |
|  | (-0.001 - 0.001) | [0.054,0.140] | [2.254,6.657] | [-0.001,0.003] | (-0.003 - 0.000) |
| Prop. carers with mental health problems | 0.00002 | 0.069*** | 3.357*** | -0.002* | -0.000 |
|  | (-0.001 - 0.001) | [0.021,0.116] | [0.992,5.722] | [-0.004,0.000] | (-0.001 - 0.001) |
| Prop. carers with a learning disability | -0.00009 | -0.04 | -1.647 | -0.004** | 0.001 |
|  | (-0.002 - 0.002) | [-0.129,0.048] | [-6.114,2.820] | [-0.007,-0.000] | (-0.002 - 0.003) |
| Prop. carers with long-standing illness | 0.00008 | -0.013 | -1.33 | -0.0004 | 0.0001 |
|  | (-0.001 - 0.001) | [-0.064,0.037] | [-3.945,1.285] | [-0.002,0.001] | (-0.001 - 0.001) |
| Prop. carers with other health conditions | 0.00001 | -0.02 | -2.092 | 0.00008 | -0.0001 |
|  | (-0.001 - 0.001) | [-0.082,0.042] | [-5.351,1.168] | [-0.002,0.002] | (-0.001 - 0.001) |
| Prop. carers with no particular health condition | 0.001 | 0.041 | 1.864 | -0.002** | 0.0004 |
|  | (-0.000 - 0.002) | [-0.013,0.095] | [-0.945,4.673] | [-0.004,-0.000] | (-0.001 - 0.002) |
| Prop. people with day-to-day activities that are limited a little | -0.691 | 252.115*** | 12302.013*** | 1.938 | -3.435* |
|  | (-3.342 - 1.960) | [106.040,398.189] | [5267.950,19336.077] | [-6.237,10.112] | (-7.126 - 0.255) |
| Prop. people with day-to-day activities that are not limited | -0.481 | 111.996** | 4566.592** | 9.411*** | -2.733** |
|  | (-2.036 - 1.074) | [18.681,205.311] | [46.575,9086.610] | [4.393,14.429] | (-5.230 - -0.236) |
| Prop. people living in house with over 0.5 and up to 1.0 persons per bedroom | -0.234 | -4.923 | -251.332 | 1.128 | -0.239 |
|  | (-0.800 - 0.332) | [-33.372,23.526] | [-1736.123,1233.459] | [-0.457,2.712] | (-0.865 - 0.386) |
| Prop. people living in house with over 1.0 and up to 1.5 persons per bedroom | -0.214 | -29.204*** | -1218.061** | -0.894 | 0.321 |
|  | (-0.676 - 0.248) | [-50.641,-7.768] | [-2325.985,-110.136] | [-2.136,0.348] | (-0.352 - 0.994) |
| Prop. people living in house with over 1.5 persons per bedroom | -0.338 | 12.178 | 634.723 | 1.03 | -0.527** |
|  | (-0.801 - 0.124) | [-9.100,33.456] | [-477.635,1747.081] | [-0.387,2.446] | (-1.048 - -0.007) |
| Prop. households with single persons aged 0-64 | -0.001 | 0.238*** | 9.344*** | 0.006 | -0.005** |
|  | (-0.004 - 0.001) | [0.114,0.362] | [2.982,15.707] | [-0.002,0.014] | (-0.009 - -0.001) |
| Prop. households with a single person aged 65 or older | 0.001 | -0.19 | -16.029* | 0.067*** | -0.004 |
|  | (-0.005 - 0.007) | [-0.531,0.151] | [-34.152,2.094] | [0.050,0.083] | (-0.012 - 0.004) |
| Prop. people who are in routine occupation | 0.003* | -0.072 | -6.26 | -0.007** | 0.003* |
|  | (-0.000 - 0.006) | [-0.221,0.076] | [-13.908,1.387] | [-0.014,-0.000] | (-0.000 - 0.006) |
| Prop. people who never worked and are long-term unemployed | 0.006* | -0.041 | -6.96 | -0.022*** | 0.008** |
|  | (-0.001 - 0.012) | [-0.330,0.248] | [-21.739,7.820] | [-0.038,-0.006] | (0.000 - 0.015) |
| Prop. people who are house owners | -0.000 | 0.038 | 1.14 | 0.005*** | -0.001* |
|  | (-0.001 - 0.001) | [-0.017,0.093] | [-1.541,3.821] | [0.002,0.009] | (-0.003 - 0.000) |
| Population density (per 10,0000 individuals) | 0.015 | 0.74 | 26.01 | -0.532*** | 0.037 |
|  | (-0.047 - 0.077) | [-2.880,4.360] | [-157.423,209.443] | [-0.743,-0.321] | (-0.033 - 0.108) |
| Population density, squared | -0.002 | -3.822*** | -194.491*** | 0.458*** | -0.002 |
|  | (-0.033 - 0.029) | [-5.357,-2.288] | [-273.199,-115.784] | [0.369,0.546] | (-0.035 - 0.030) |
| Prop. people aged 18-64 entitled to disability support | -0.002 | 0.115 | 8.621 | 0.046** | -0.009 |
|  | (-0.017 - 0.013) | [-0.628,0.858] | [-28.139,45.382] | [0.002,0.089] | (-0.027 - 0.009) |
| Prop. people aged 65 or older entitled to disability support | -0.010 | 2.759*** | 116.808*** | 0.053 | -0.045** |
|  | (-0.035 - 0.015) | [1.330,4.188] | [48.884,184.733] | [-0.038,0.144] | (-0.084 - -0.006) |
| Prop. people aged 65 or older claiming attendance allowance | -0.000 | 0.591*** | 32.148*** | -0.016*** | -0.004* |
|  | (-0.003 - 0.003) | [0.452,0.731] | [25.152,39.144] | [-0.024,-0.008] | (-0.008 - 0.000) |
| Prop. people receiving some form of income support | -0.003 | -0.480* | -32.084*** | 0.050*** | -0.006 |
|  | (-0.013 - 0.006) | [-0.977,0.018] | [-56.178,-7.991] | [0.027,0.074] | (-0.017 - 0.005) |
| Prop. people aged 18-64 are entitled to employment allowance | 0.005 | 0.151 | 7.91 | 0.045*** | -0.001 |
|  | (-0.002 - 0.012) | [-0.272,0.575] | [-13.736,29.555] | [0.023,0.066] | (-0.010 - 0.009) |
| Prop. people aged 18 or older entitled to Personal Independence Payment | -0.000 | -0.077 | -10.916 | 0.076*** | -0.010 |
|  | (-0.011 - 0.011) | [-0.588,0.434] | [-36.484,14.652] | [0.048,0.104] | (-0.025 - 0.005) |
| Index of multiple deprivation: quartile 2 | -0.001 | 0.338 | 15.093 | 0.034*** | -0.007 |
|  | (-0.014 - 0.012) | [-0.321,0.998] | [-16.638,46.825] | [0.011,0.057] | (-0.022 - 0.009) |
| Index of multiple deprivation: quartile 3 | -0.007 | 0.133 | 4.452 | 0.009 | -0.012 |
|  | (-0.026 - 0.012) | [-0.766,1.033] | [-40.457,49.361] | [-0.032,0.049] | (-0.033 - 0.010) |
| Index of multiple deprivation: quartile 4 (most deprived) | -0.016 | 0.637 | 36.138 | -0.019 | -0.019 |
|  | (-0.040 - 0.009) | [-0.639,1.914] | [-24.744,97.019] | [-0.074,0.035] | (-0.047 - 0.010) |
| Index of education deprivation: quartile 2 | -0.011* | -0.354 | -16.2 | -0.094*** | 0.003 |
|  | (-0.021 - 0.000) | [-0.899,0.191] | [-45.320,12.920] | [-0.118,-0.070] | (-0.013 - 0.019) |
| Index of education deprivation: quartile 3 | -0.012 | -0.854*** | -37.107** | -0.137*** | 0.014 |
|  | (-0.027 - 0.003) | [-1.497,-0.211] | [-70.535,-3.680] | [-0.170,-0.103] | (-0.012 - 0.039) |
| Index of education deprivation: quartile 4 (most deprived) | -0.018* | -0.322 | -17.797 | -0.191*** | 0.005 |
|  | (-0.037 - 0.001) | [-1.211,0.567] | [-63.328,27.733] | [-0.238,-0.145] | (-0.021 - 0.032) |
| Index of income deprivation: quartile 2 | -0.00009 | 0.141 | 6.021 | 0.047*** | -0.009 |
|  | (-0.014 - 0.014) | [-0.521,0.803] | [-28.477,40.520] | [0.019,0.075] | (-0.025 - 0.007) |
| Index of income deprivation: quartile 3 | -0.007 | 0.327 | 19.714 | 0.071*** | -0.014 |
|  | (-0.028 - 0.014) | [-0.713,1.368] | [-34.711,74.139] | [0.025,0.116] | (-0.039 - 0.010) |
| Index of income deprivation: quartile 4 (most deprived) | -0.003 | 1.552** | 85.606** | 0.112*** | -0.027 |
|  | (-0.029 - 0.023) | [0.147,2.956] | [14.523,156.690] | [0.054,0.170] | (-0.064 - 0.009) |
| Index of employment deprivation: quartile 2 | -0.001 | 0.026 | 1.075 | -0.050*** | 0.004 |
|  | (-0.014 - 0.012) | [-0.643,0.695] | [-31.275,33.425] | [-0.076,-0.024] | (-0.010 - 0.019) |
| Index of employment deprivation: quartile 3 | -0.007 | 0.808 | 42.429* | -0.088*** | -0.004 |
|  | (-0.025 - 0.012) | [-0.172,1.787] | [-5.461,90.319] | [-0.128,-0.048] | (-0.025 - 0.017) |
| Index of employment deprivation: quartile 4 (most deprived) | -0.009 | -0.159 | -7.864 | -0.060** | 0.003 |
|  | (-0.033 - 0.015) | [-1.362,1.043] | [-67.674,51.945] | [-0.117,-0.002] | (-0.025 - 0.030) |
| Index of health and disability deprivation: quartile 2 | 0.003 | -0.203 | 0.088 | -0.014 | 0.009 |
|  | (-0.007 - 0.014) | [-0.726,0.320] | [-27.838,28.013] | [-0.035,0.008] | (-0.005 - 0.023) |
| Index of health and disability deprivation: quartile 3 | 0.010 | 0.298 | 20.504 | -0.104*** | 0.019* |
|  | (-0.006 - 0.025) | [-0.446,1.043] | [-19.234,60.241] | [-0.137,-0.072] | (-0.001 - 0.038) |
| Index of health and disability deprivation: quartile 4 (most deprived) | 0.012 | 0.532 | 30.03 | -0.115*** | 0.020* |
|  | (-0.007 - 0.032) | [-0.475,1.539] | [-22.781,82.841] | [-0.157,-0.073] | (-0.003 - 0.043) |
| Constant | 1.211 | -129.18** | -5,685.5** | -4.29 | 2.991** |
|  | (-0.459 - 2.880) | (-229.0 - -29.36) | (-10,522.18 - -8,48.8) | (-9.97- 1.4) | (0.623 - 5.359) |
| Year fixed effects | Yes | Yes | Yes | Yes | Yes |
| Region fixed effects | Yes | Yes | Yes | Yes | Yes |
| Interaction year and region fixed effects | Yes | Yes | Yes | Yes | Yes |
| Local authority fixed effects | No | No | No | No | No |
| Observations | 332,859 | 332,859 | 332,859 | 332,859 | 332,859 |
| Coverage distortion |  |  | |  | 5% |
| ASC=Adult Social Care. OLS= ordinary least squares. 95% confidence intervals in parentheses  A coverage distortion greater than 10% indicates that the instruments are overall weak. The coverage distortion in regression (1) is obtained using a grid of 1,685 coefficients including 21 coefficients for ASC expenditure per user, 1 coefficient for ASC expenditure per user squared, and 78 coefficients for the proportions of users. In regression (2), the coverage distortion is obtained using an interval of 1,000 coefficients for ASC expenditure per client. Observations are weighted by the survey weight and standard errors are clustered within strata and local authorities.  ***=p-value<0.01, **=p-value<0.05, *=p-value<0.1 | | | | | |

Table A4b – Full results from regression (2) estimated by OLS and 2SLS

| Variable | **Regression (2)** | | |
| --- | --- | --- | --- |
|  | OLS | 2SLS | |
|  |  | First stage | Second stage |
|  |  |  |  |
| ASC expenditure per LTS user at mean level | 0.00004 |  | 0.002** |
|  | (-0.000 - 0.001) |  | (0.000 - 0.003) |
| ASC expenditure per LTS user, squared |  |  |  |
|  |  |  |  |
| Prop. of LTS users |  |  |  |
| Council tax base per user at zero level |  |  |  |
|  |  |  |  |
| Council tax base per user, squared |  |  |  |
|  |  |  |  |
| County authority |  |  |  |
|  |  |  |  |
| Metropolitan authority |  |  |  |
|  |  |  |  |
| Unitary authority |  |  |  |
|  |  |  |  |
| Missing council tax revenues per user |  | 0.004*** |  |
|  |  | [0.003,0.005] |  |
| Missing council tax revenues per user, squared |  | -0.00000*** |  |
|  |  | [-0.000,-0.000] |  |
| Female | -0.003** | 0.013* | -0.003** |
|  | (-0.006 - -0.001) | [-0.002,0.028] | (-0.006 - -0.001) |
| Aged 65 or older | 0.039*** | 0.01 | 0.039*** |
|  | (0.034 - 0.045) | [-0.018,0.037] | (0.034 - 0.044) |
| White ethnicity | 0.029*** | 0.048** | 0.029*** |
|  | (0.025 - 0.033) | [0.003,0.093] | (0.025 - 0.033) |
| Questionnaire in English | 0.052*** | 0.143* | 0.052*** |
|  | (0.023 - 0.081) | [-0.011,0.298] | (0.023 - 0.081) |
| No help with the questionnaire | 0.017*** | -0.017 | 0.017*** |
|  | (0.012 - 0.022) | [-0.044,0.009] | (0.012 - 0.022) |
| The questionnaire was read by someone else | 0.035*** | -0.01 | 0.035*** |
|  | (0.032 - 0.038) | [-0.029,0.009] | (0.032 - 0.038) |
| The questionnaire’s questions were translated by someone else | 0.003** | 0.034** | 0.003** |
|  | (0.000 - 0.006) | [0.007,0.061] | (0.000 - 0.006) |
| The questionnaire’s answers were written by someone else | -0.015*** | -0.001 | -0.015*** |
|  | (-0.018 - -0.013) | [-0.018,0.017] | (-0.018 - -0.013) |
| The questionnaire’s questions were talked through with someone else | -0.017*** | -0.022*** | -0.017*** |
|  | (-0.019 - -0.015) | [-0.037,-0.006] | (-0.019 - -0.014) |
| The questionnaire’s questions were answered by someone else without asking | -0.066*** | 0.028* | -0.066*** |
|  | (-0.070 - -0.061) | [-0.001,0.057] | (-0.070 - -0.061) |
| Easy-read version of the questionnaire | 0.046*** | 0.286* | 0.045*** |
|  | (0.028 - 0.063) | [-0.015,0.588] | (0.028 - 0.063) |
| Sensory support | -0.003 | -0.115** | -0.003 |
|  | (-0.011 - 0.005) | [-0.221,-0.009] | (-0.011 - 0.005) |
| Support with memory and cognition | 0.030*** | 0.027 | 0.030*** |
|  | (0.024 - 0.035) | [-0.027,0.081] | (0.024 - 0.035) |
| Learning disability support | 0.100*** | -0.254* | 0.100*** |
|  | (0.083 - 0.117) | [-0.545,0.037] | (0.083 - 0.117) |
| Mental health support | 0.013*** | -0.051* | 0.013*** |
|  | (0.007 - 0.020) | [-0.102,0.001] | (0.007 - 0.020) |
| Social support | 0.008* | -0.021 | 0.008* |
|  | (-0.001 - 0.017) | [-0.092,0.050] | (-0.001 - 0.017) |
| Prop. carers who are female | 0.00008 | -0.058*** | 0.0001 |
|  | (-0.000 - 0.001) | [-0.094,-0.022] | (-0.000 - 0.001) |
| Prop. carers aged 65 or older | -0.00001 | -0.020* | -0.000003 |
|  | (-0.000 - 0.000) | [-0.042,0.001] | (-0.000 - 0.000) |
| Prop. carers who are white | -0.00002 | 0.007 | -0.00003 |
|  | (-0.000 - 0.000) | [-0.004,0.017] | (-0.000 - 0.000) |
| Prop. carers who are retired | -0.0002 | -0.039 | 2 |
|  | (-0.001 - 0.000) | [-0.097,0.019] | (-0.001 - 0.000) |
| Prop. carers who are employed full-time | -0.001* | -0.012 | -0.001* |
|  | (-0.002 - 0.000) | [-0.096,0.071] | (-0.002 - 0.000) |
| Prop. carers who are employed part-time | -0.0006 | -0.083** | -0.001 |
|  | (-0.002 - 0.000) | [-0.155,-0.011] | (-0.002 - 0.000) |
| Prop. carers who are self-employed full-time | 0.001 | -0.139* | 0.001 |
|  | (-0.000 - 0.003) | [-0.282,0.004] | (-0.000 - 0.003) |
| Prop. carers who are self-employed part-time | 0.001 | -0.002 | 0.001 |
|  | (-0.000 - 0.002) | [-0.112,0.108] | (-0.001 - 0.002) |
| Prop. carers who are not in paid work | -0.00009 | 0.057* | -0.0005 |
|  | (-0.001 - 0.001) | [-0.002,0.115] | (-0.001 - 0.000) |
| Prop. carers who are doing voluntary work | -0.0005 | 0.103** | -0.001 |
|  | (-0.001 - 0.001) | [0.009,0.197] | (-0.002 - 0.000) |
| Prop. carers who are doing other type of work | -0.0005 | -0.053 | -0.001 |
|  | (-0.002 - 0.001) | [-0.141,0.036] | (-0.002 - 0.001) |
| Prop. carers with physical impairment or disability | -0.0006 | 0.022 | -0.0004 |
|  | (-0.001 - 0.000) | [-0.023,0.067] | (-0.001 - 0.000) |
| Prop. carers with sight or hearing loss | -0.0003 | 0.015 | -0.00005 |
|  | (-0.001 - 0.000) | [-0.034,0.064] | (-0.001 - 0.000) |
| Prop. carers with mental health problems | -0.00001 | -0.059* | 0.00008 |
|  | (-0.001 - 0.001) | [-0.124,0.006] | (-0.001 - 0.001) |
| Prop. carers with a learning disability | -0.0005 | -0.009 | -0.0004 |
|  | (-0.002 - 0.001) | [-0.106,0.087] | (-0.002 - 0.001) |
| Prop. carers with long-standing illness | -0.00005 | 0.012 | -0.00005 |
|  | (-0.001 - 0.001) | [-0.042,0.065] | (-0.001 - 0.001) |
| Prop. carers with other health conditions | -0.0004 | -0.068* | -0.0002 |
|  | (-0.001 - 0.000) | [-0.139,0.002] | (-0.001 - 0.000) |
| Prop. carers with no particular health condition | 0.0001 | -0.055 | 0.0002 |
|  | (-0.001 - 0.001) | [-0.125,0.015] | (-0.000 - 0.001) |
| Prop. people with day-to-day activities that are limited a little | -7.035 | 3100.462*** | -9.953 |
|  | (-22.890 - 8.820) | [2207.565,3993.359] | (-26.158 - 6.253) |
| Prop. people with day-to-day activities that are not limited | -3.731 | 673.871*** | -4.289 |
|  | (-10.249 - 2.788) | [257.972,1089.771] | (-10.867 - 2.289) |
| Prop. people living in house with over 0.5 and up to 1.0 persons per bedroom | -2.325 | 8.397 | -3.100 |
|  | (-8.177 - 3.527) | [-423.918,440.712] | (-9.038 - 2.838) |
| Prop. people living in house with over 1.0 and up to 1.5 persons per bedroom | 0.651 | 2002.104*** | -1.798 |
|  | (-5.291 - 6.594) | [1626.408,2377.799] | (-8.339 - 4.743) |
| Prop. people living in house with over 1.5 persons per bedroom | -4.314** | 188.234 | -4.822** |
|  | (-8.263 - -0.365) | [-129.422,505.891] | (-8.828 - -0.815) |
| Prop. households with single persons aged 0-64 | -0.008 | 0.735 | -0.008 |
|  | (-0.023 - 0.006) | [-0.269,1.738] | (-0.023 - 0.006) |
| Prop. households with a single person aged 65 or older | 0.002 | 3.639*** | -0.002 |
|  | (-0.022 - 0.026) | [1.669,5.608] | (-0.026 - 0.022) |
| Prop. people who are in routine occupation | -0.005 | -1.895* | -0.005 |
|  | (-0.035 - 0.025) | [-4.117,0.327] | (-0.035 - 0.025) |
| Prop. people who never worked and are long-term unemployed | -0.022 | -1.014 | -0.018 |
|  | (-0.054 - 0.010) | [-3.728,1.699] | (-0.051 - 0.014) |
| Prop. people who are house owners | -0.006 | -0.308 | -0.005 |
|  | (-0.017 - 0.005) | [-1.004,0.388] | (-0.016 - 0.006) |
| Population density (per 10,0000 individuals) | 0.378 | -145.222*** | 0.592** |
|  | (-0.108 - 0.863) | [-205.923,-84.522] | (0.035 - 1.149) |
| Population density, squared | -0.096 | 44.877*** | -0.167* |
|  | (-0.271 - 0.079) | [23.911,65.843] | (-0.364 - 0.029) |
| Prop. people aged 18-64 entitled to disability support | 0.004 | -1.857*** | 0.006 |
|  | (-0.010 - 0.017) | [-3.084,-0.631] | (-0.008 - 0.019) |
| Prop. people aged 65 or older entitled to disability support | -0.010 | 3.610*** | -0.015 |
|  | (-0.032 - 0.011) | [1.562,5.657] | (-0.038 - 0.008) |
| Prop. people aged 65 or older claiming attendance allowance | 0.003 | 0.369* | 0.002 |
|  | (-0.002 - 0.007) | [-0.012,0.749] | (-0.002 - 0.007) |
| Prop. people receiving some form of income support | 0.002 | 0.568 | 0.001 |
|  | (-0.007 - 0.012) | [-0.380,1.516] | (-0.009 - 0.011) |
| Prop. people aged 18-64 are entitled to employment allowance | 0.000 | -0.134 | 0.001 |
|  | (-0.004 - 0.005) | [-0.607,0.338] | (-0.004 - 0.006) |
| Prop. people aged 18 or older entitled to Personal Independence Payment | 0.003 | -0.517 | 0.004 |
|  | (-0.007 - 0.012) | [-1.410,0.376] | (-0.006 - 0.013) |
| Index of multiple deprivation: quartile 2 | -0.004 | -1.126*** | -0.003 |
|  | (-0.015 - 0.006) | [-1.712,-0.539] | (-0.014 - 0.008) |
| Index of multiple deprivation: quartile 3 | -0.010 | -3.527*** | -0.004 |
|  | (-0.025 - 0.005) | [-5.402,-1.652] | (-0.021 - 0.012) |
| Index of multiple deprivation: quartile 4 (most deprived) | -0.023** | -3.028*** | -0.018* |
|  | (-0.040 - -0.005) | [-4.981,-1.075] | (-0.037 - 0.001) |
| Index of education deprivation: quartile 2 | -0.008 | 0.49 | -0.009* |
|  | (-0.018 - 0.002) | [-0.102,1.082] | (-0.019 - 0.001) |
| Index of education deprivation: quartile 3 | -0.013 | 0.932** | -0.015* |
|  | (-0.029 - 0.004) | [0.187,1.677] | (-0.031 - 0.002) |
| Index of education deprivation: quartile 4 (most deprived) | -0.014 | 1.040** | -0.016* |
|  | (-0.032 - 0.004) | [0.045,2.035] | (-0.034 - 0.002) |
| Index of income deprivation: quartile 2 | -0.004 | 0.771*** | -0.006 |
|  | (-0.014 - 0.005) | [0.186,1.356] | (-0.016 - 0.004) |
| Index of income deprivation: quartile 3 | -0.009 | 2.739*** | -0.013 |
|  | (-0.024 - 0.007) | [1.563,3.914] | (-0.029 - 0.004) |
| Index of income deprivation: quartile 4 (most deprived) | 0.002 | 2.769*** | -0.002 |
|  | (-0.018 - 0.022) | [1.371,4.167] | (-0.022 - 0.019) |
| Index of employment deprivation: quartile 2 | 0.002 | 0.667** | 0.001 |
|  | (-0.009 - 0.013) | [0.005,1.328] | (-0.010 - 0.011) |
| Index of employment deprivation: quartile 3 | 0.005 | 0.968** | 0.003 |
|  | (-0.008 - 0.018) | [0.030,1.906] | (-0.010 - 0.016) |
| Index of employment deprivation: quartile 4 (most deprived) | 0.006 | 1.325* | 0.004 |
|  | (-0.011 - 0.022) | [-0.017,2.668] | (-0.013 - 0.020) |
| Index of health and disability deprivation: quartile 2 | 0.004 | 0.15 | 0.004 |
|  | (-0.004 - 0.013) | [-0.618,0.918] | (-0.005 - 0.012) |
| Index of health and disability deprivation: quartile 3 | 0.010* | 0.264 | 0.009 |
|  | (-0.001 - 0.020) | [-1.105,1.634] | (-0.002 - 0.020) |
| Index of health and disability deprivation: quartile 4 (most deprived) | 0.010 | -0.721 | 0.010 |
|  | (-0.003 - 0.023) | [-2.248,0.806] | (-0.003 - 0.024) |
| Constant | 6.593 | -1,293.3*** | 8.232* |
|  | (-2.810 - 15.995) | (-1,876.6 - -710) | (-1.363 - 17.828) |
| Year fixed effects | Yes | Yes | Yes |
| Region fixed effects | No | No | No |
| Interaction year and region fixed effects | No | No | No |
| Local authority fixed effects | Yes | Yes | Yes |
| Observations | 332,859 | 332,281 | 332,281 |
| Coverage distortion |  |  | 5% |
| ASC=Adult Social Care. OLS= ordinary least squares. 95% confidence intervals in parentheses  A coverage distortion greater than 10% indicates that the instruments are overall weak. The coverage distortion in regression (1) is obtained using a grid of 1,685 coefficients including 21 coefficients for ASC expenditure per user, 1 coefficient for ASC expenditure per user squared, and 78 coefficients for the proportions of users. In regression (2), the coverage distortion is obtained using an interval of 1,000 coefficients for ASC expenditure per client. Observations are weighted by the survey weight and standard errors are clustered within strata and local authorities.  ***=p-value<0.01, **=p-value<0.05, *=p-value<0.1 |  |  |  |

Table A5 – Hypothesis tests of differences between coefficients from the analysis of user groups.

|  | Users (b) vs (a) | Users (b) vs (c) | Users (b) vs (d) |
| --- | --- | --- | --- |
| Difference in marginal effects at mean expenditure | p-value=0.071* | p-value=0.058* | p-value=0.498 |
| Users (a)=users with a learning disability, (b)=users with no learning disability aged between 16-64 , (c)=users with no learning disability aged 65 or older in residential or nursing care , (d)=users with no learning disability aged 65 or older using community-based care. | | | |
| Wald tests for linear hypotheses after estimation were used to test for differences in marginal effects at the mean level of ASC expenditure across groups of users. This implies testing the same regression but on different samples. | | | |
| ***=p-value<0.01, **=p-value<0.05, *=p-value<0.1 | | | |

Table A6 – Results from the LA fixed effects analysis of user groups and CRQoL domains.

| **User groups** | | | | |
| --- | --- | --- | --- | --- |
|  | (a) | (b) | (c) | (d) |
| Marginal effect of ASC expenditure per user | -0.0012 | 0.0024 | -0.0005 | 0.0035*** |
| Observations | 86,668 | 64,361 | 55,179 | 126,073 |
| **CRQoL domains** | | | | |
|  | Personal cleanliness | Accommodation cleanliness | Food and drink | Safety |
| Marginal effect of ASC expenditure per user | 0.0007 | -0.00001 | 0.00003 | 0.0004** |
| Observations | 367,158 | 365,927 | 364,254 | 366,372 |
|  | Social participation | Occupation | Control over daily life | Dignity |
| Marginal effect of ASC expenditure per user | 0.0003** | 0.0004** | 0.0003* | 0.0003* |
| Observations | 365,168 | 362,294 | 366,410 | 358,327 |
| ASC=Adult Social Care, CRQoL-Care-Related Quality of Life | | | | |
| User group (a)=users with a learning disability, (b)=users with no learning disability aged between 16-64 , (c)=users with no learning disability aged 65 or older in residential or nursing care , (d)=users with no learning disability aged 65 or older using community-based care. | | | | |
| The controls and instruments in these regressions are the same as in (2), as well as observations are weighted by the survey weight and standard errors are clustered within strata and local authorities. | | | | |
| ***=p-value<0.01, **=p-value<0.05, *=p-value<0.1 | | | | |

# References

HOUTHAKKER, H. S. 1965. New evidence on demand elasticities. *Econometrica: Journal of the Econometric Society***,** 277-288.

KUH, E. 1959. The validity of cross-sectionally estimated behavior equations in time series applications. *Econometrica: Journal of the Econometric Society***,** 197-214.

LOCAL GOVERNMENT ASSOCIATION. 2017. *How is £1 of your council tax spent?* [Online]. Available: <https://www.local.gov.uk/sites/default/files/documents/A4%20STATIC%20IMAGE_04_1.pdf> [Accessed 25 January 2021].

1. This is despite freeze grants being a permanent part of the funding that every year the central government transfers to LAs, except for the one-off freeze grant in 2012/13. [↑](#footnote-ref-1)
